# Inactivating histone deacetylase HDA promotes longevity by mobilizing trehalose metabolism

**DOI:** 10.1038/s41467-021-22257-2

**Published:** 2021-03-31

**Authors:** Ruofan Yu, Xiaohua Cao, Luyang Sun, Jun-yi Zhu, Brian M Wasko, Wei Liu, Emeline Crutcher, Haiying Liu, Myeong Chan Jo, Lidong Qin, Matt Kaeberlein, Zhe Han, Weiwei Dang

**Affiliations:** 1 Department of Molecular and Human Genetics, Baylor College of Medicine, Houston, TX USA; 2 Huffington Center on Aging, Baylor College of Medicine, Houston, TX USA; 3 Center for Precision Disease Modeling, University of Maryland School of Medicine, Baltimore, MD USA; 4 Department of Pathology, University of Washington, Seattle, WA USA; 5 Innovative BioChips, Sugar Land, TX USA; 6 Department of Nanomedicine, Houston Methodist Research Institute, Houston, TX USA; 7 Present Address: University of Houston, Clear Lake, TX USA

**Keywords:** Stress signalling, Acetylation, Ageing, Epigenetics

## Abstract

Histone acetylations are important epigenetic markers for transcriptional activation in response to metabolic changes and various stresses. Using the high-throughput SEquencing-Based Yeast replicative Lifespan screen method and the yeast knockout collection, we demonstrate that the HDA complex, a class-II histone deacetylase (HDAC), regulates aging through its target of acetylated H3K18 at storage carbohydrate genes. We find that, in addition to longer lifespan, disruption of HDA results in resistance to DNA damage and osmotic stresses. We show that these effects are due to increased promoter H3K18 acetylation and transcriptional activation in the trehalose metabolic pathway in the absence of HDA. Furthermore, we determine that the longevity effect of HDA is independent of the Cyc8-Tup1 repressor complex known to interact with HDA and coordinate transcriptional repression. Silencing the HDA homologs in *C. elegans* and *Drosophila* increases their lifespan and delays aging-associated physical declines in adult flies. Hence, we demonstrate that this HDAC controls an evolutionarily conserved longevity pathway.

## Abstract (web summary)

Histone acetylations are important epigenetic marks for transcriptional activation and respond to metabolic changes. Here the authors develop a lifespan screen and show that inactivation of the histone deacetylase complex activates longevity and protects against stress via trehalose metabolism.

## Introduction

Aging is a complex biological phenomenon associated with functional declines and accumulated damages or abnormalities at all levels of an organism, from organs and tissues to cellular macromolecules^1^. Understanding the molecular underpinning of aging is vital to intervene against human health risks, because aging is a major risk for numerous human diseases that are the principal cause of death. The single-celled budding yeast *Saccharomyces cerevisiae* is an important model for cellular and organismal aging due to its short lifespan and the power of its available genetic tools. Several longevity-associated molecular pathways, such as TOR signaling and sirtuin activities, have been identified and extensively characterized using yeast, and these evolutionarily conserved pathways have been invaluable to the biological understanding of aging^2,3^. Continued study of the molecular mechanisms that affect yeast lifespan may lead to treatments for age-related diseases in humans and delay the onset of aging.

Yeast replicate by asymmetrical cell division. In each cell cycle event, a small daughter cell, referred to as a bud, separates from the mother. One yeast cell can undergo a limited number of cell divisions before entering a permanently arrested state. This replication capacity is called replicative lifespan (RLS). Traditionally, measuring RLS involves physically separating daughter cells from their mothers on agar plates, using a manual micromanipulator equipped with a fiber-optic needle^4,5^. Because of extensive labor required, an RLS screen using a yeast knockout collection was only recently completed using manual microdissection^6^. Thanks to the tremendous effort made by the authors, several important and conserved aging regulating pathways were identified through this screen, such as ribosome biogenesis^7^. However, due to the amount of work required to perform such experiments, data were only obtained from five individual cells in the majority of strains. This tedious and low-throughput experimental method is not compatible with routine screens of yeast mutant collections, which normally contain thousands of strains. Thus, the limitations of traditional screening methods hinder the progress of aging research.

Recently, several groups developed microfluidic-based methods to tackle this problem^8–12^. Microfluid-based technology allows significantly shorter experiment times, from 3–4 weeks to merely 3 days, yet the throughput of this method is still limited by the necessity to manually count cell division times. Therefore, strategies are required to take advantage of the power of yeast genetics and apply unbiased and high-throughput genetic screening approaches to yeast aging research^12^. To date, there have been few advancements on this front. One example, the High-Life technique is based on mother cell enrichment and flow-cytometry^13^; however, due to special requirements of the yeast genotype during mother cell enrichment, this method is more suitable for discovering lifespan-affecting chemical compounds in a limited number of yeast strain genotypes. Another screening method developed by Sen et al. utilized old cell sorting techniques and an oligonucleotide chip array of a barcoded histone mutant collection; however, the method provided no statistical comparison with traditional low-throughput methods, and the barcode microarray did not have a dynamic range or throughput comparable to sequencing^14^. Therefore, we believe that developing and carefully validating high-throughput yeast RLS screening methods is vitally important. Ideally, such methods would be based on accurate quantification and be easy to apply on different types of yeast mutant collections.

In this work, we set out to develop a high-throughput screening method, SEquencing-Based Yeast replicative Lifespan screen (SEBYL), to identify previously unknown aging regulators in budding yeast. By using SEBYL with a yeast knockout collection, we identified 285 long-lived gene deletions. Notably, a significant portion of these had extended lifespans in previous classical experiments. To demonstrate our method’s ability to discover previously unidentified genes and pathways involved in aging, we focused on characterizing one aging regulator that was identified from the screening, histone deacetylase complex HDA. We found that HDA regulates aging via stress response pathways, particularly in the DNA damage stress response. The presence of the HDA complex inhibits expression of trehalose metabolism genes, which protect against stress. When the HDA complex is mutated, trehalose genes are de-repressed, enhancing the stress response, and eventually promoting longevity. In summary, SEBYL is a robust method that saves time and energy and can discover aging regulating genes using various preexisting yeast mutant collection resources.

## Results

### The SEBYL experimental pipeline

**Fig. 1:**
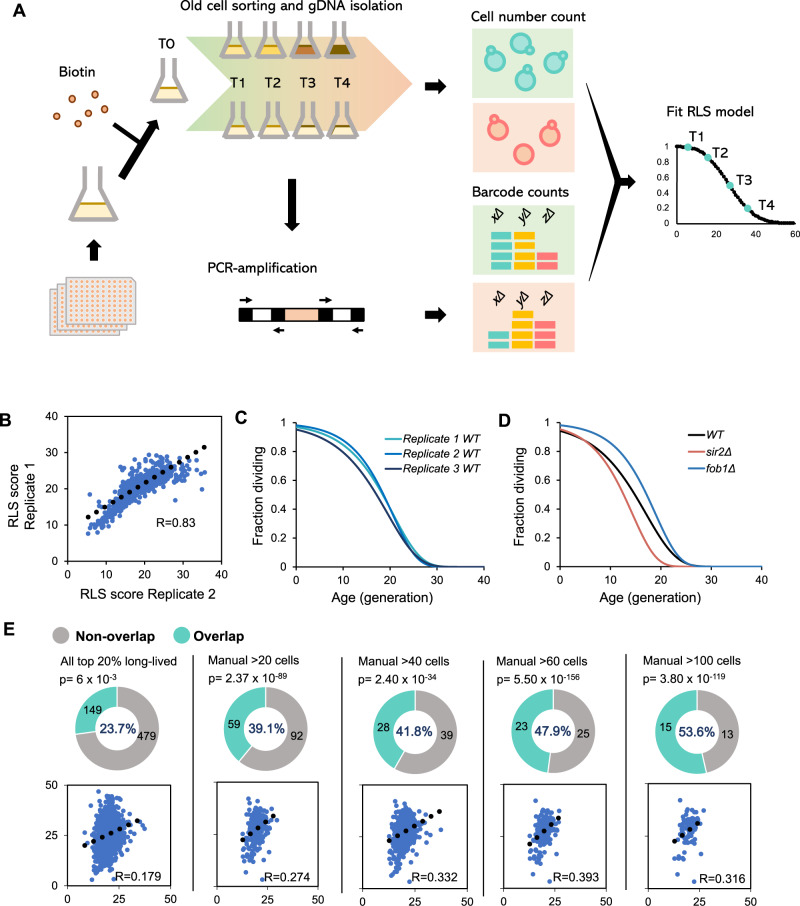
Pipeline and quality control for SEBYL method. **A** Pipeline of SEBYL screening method. **B** Correlation analysis of final RLS score from screening replicate 1 and replicate 2. **C** Survival curves of wild-type BY4742 strains from three replicates. **D** Survival curves of wild-type, *sir2Δ* and *fob1Δ* strains. **E** Overlap between long-lived yeast deletion strains identified from SEBYL and manual microdissection screening. Manual results were grouped by number of cells included in experiments. Bottom figures indicate correlations between manual and SEBYL results. *X*-axis indicates SEBYL RLS score, *Y*-axis indicates RLS measured by manual screen. *p*-value calculated by *χ*^2^ test. See also Fig. S1.

We developed an improved high-throughput yeast RLS screen method, SEBYL, based on an existing protocol^15^. First, all strains in a barcoded yeast mutant collection were pooled together, labeled with NHS-LC-biotin, and cultured overnight. Four consecutive rounds of old cell purification experiments harvested cells with advanced replicative age (Fig. 1A). Over successive sorting of old mother cells, long-lived mutants are expected to be enriched in old fractions, whereas short-lived mutants will be depleted, as reported previously^15^. To account for growth defects and improve the accuracy of lifespan predictions, young cell populations are also collected at each sorting for barcode sequencing to determine cell cycle times for each mutant during each growth period. We utilized this improved screening design with the yeast knockout (YKO) collection that contains 4828 viable single gene deletion mutants. In this library, each individual gene is replaced by the selection marker kanMX4, which is flanked by unique UPTAG and DNTAG barcode sequences. We collected sorted fractions of young and old cells from the first, second, third, and fourth rounds of sorting, as well as an unsorted fraction, for analysis. The unsorted fraction represents the initial relative distribution of all the mutants in the pool. Whereas the relative enrichment for each mutant in the old cell fractions is influenced by both age and growth rate, the relative enrichment in the young cell fractions reflect only the growth rate. Hence, incorporating both young and old cell fractions in the lifespan modeling can accurately estimate relative difference in lifespan. The YKO lifespan screen was performed in three biological replicates. For each sorting, we (1) counted the number of cells present in each sample, and (2) extracted genomic DNA from each sample and performed barcode sequencing analysis to determine the relative abundance of each mutant strain. A modified Gompertz nonlinear regression model was used to estimate RLS for 3437 deletion strains from the YKO library.

### SEBYL yields reproducible high-quality results

First, we validated the repeatability of three parallel experiments by calculating the correlation coefficient of barcode counts between each biological replicate. We confirmed high correlation in populations ranging from youngest (S1O) to oldest (S4O; Fig. S1A). Even after incorporating all nine data points and extensive modeling, the correlation coefficients for calculated RLS scores were still reasonable, in the range of 0.75–0.83 among all three biological replicates, especially when considering the numerous complicated cell sorting steps in each of the week-long screening experiments. Our data consistency is either comparable or better than the reported chronological lifespan screens^16,17^ (Figs. 1B and S1B, S1C). The wild-type strain had very similar lifespan curves across three experiments (Fig. 1C), and two control strains included in the screen, short-lived *sir2Δ* and long-lived *fob1Δ*, both showed the expected lifespan phenotypes (Fig. 1D). From these data, we conclude that the screen is reproducible and technically sound.

### Lifespan estimation by SEBYL is concordant with published data

Because a previous RLS screening of the YKO library was performed by manual dissection^6^, we can comprehensively assess the accuracy of SEBYL by comparing our results to the manual method. Due to the enormous amount of time and effort required to perform a manual screen, McCormick et al.^6^ designed the experiment to start with five mutant cells and measured the RLS of additional cells only after data from the initial five cells in the manual screen yielded statistically significant results. As such, RLS data from the majority of mutant strains (~70%) remained inconclusive. Presumably, the inclusion of more mother cells in the manual RLS measurement would decrease the false positive and false negative discovery rates, so that the measured lifespan would approach the actual population average^6^. We therefore categorized manual screening results by the number of mother cells that were counted in the experiments and compared how many long-lived mutants (defined as the top 20% longest RLS) were present in both manual and SEBYL datasets. When we included all available mutants in the manual screen, we found 23% overlap. But as cell number increased, the proportion of overlap increased drastically, reaching 53.6% when only including strains that had more than 100 cells assessed for RLS in manual screen (Fig. 1E).

We then took the long-lived and short-lived mutants identified by manual screening and determined how they ranked in our experiment. As expected, long-lived strains were enriched in the highest-ranking fractions of SEBYL results (Fig. S1D), whereas the opposite was true for short-lived strains (Fig. S1E). Additionally, we performed the same analysis using the RLS dataset from the *Saccharomyces* Genome Database^18^, which includes results from both high-throughput and classical experiments, and we observed a similar enrichment of long-lived strains in the fraction that contained the highest-ranking mutants (Fig. S1F). We believe these results confirm the ability of SEBYL to yield RLS data comparable to manual measurements.

### Age-related morphological changes

**Fig. 2:**
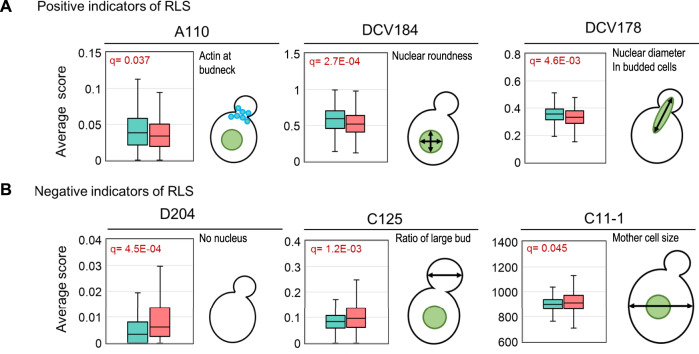
Examples of age-related morphology changes. Positive (**A**) and negative (**B**) indicators of RLS are shown with distribution of average score of designated morphology traits, calculated from long-lived (green) and short-lived (red) groups. Box blots center represents median, whisker represents 1.5 inter quartile range, and lower and upper hinges represent first and third quartiles, outliers were removed for clarity.

We obtained RLS data for 3437 deletion strains, 2294 of which did not have conclusive RLS results from previous manual screening (<5 cells counted). SEBYL results can be used for functional analysis of preexisting high-throughput datasets to identify or characterize aging-related factors. Yeast cell morphology is a general indication of their well-being, and a previous study indicated that cell size is negatively correlated with RLS^19^. Thus, we wanted to see if other yeast morphological traits could indicate RLS. We took advantage of the *Saccharomyces cerevisiae* Morphological Database^20^, which contains comprehensive quantitative records for morphological features of all available YKO strains. We compared each morphological trait between top the 10% and bottom 10% of long-lived strains to see if the average scores were significantly different (see Supplementary Data 1 for complete analysis results). A few traits emerged as positive indicators of longevity and three examples are: actin localized at bud site (A110), nuclear roundness (DCV184), and nuclear diameter in budded cells (DCV178; Fig. 2A). Accumulation of actin at the bud neck and increased nuclear diameter are indications that cells are actively undergoing cell division. Although the biological significance of nuclear roundness is not entirely understood, a deformed nucleus usually indicates that cells are not healthy.

For negative indicators of RLS, we identified several traits associated with nuclear abnormality as well as cell size, such as having no nuclei in the cell (D204), large bud size (C125), or large mother cell size (C11–1; Fig. 2B). An altered number of nuclei potentially indicates polyploidy and aneuploidy, which are detrimental for the normal aging process^21^. Abnormal nucleus number could also result from mis-segregation, which becomes more evident as cells undergo replicative aging^22^. A larger size for either daughter or mother cells is also suggested to negatively correlate with lifespan, in agreement with previous reports^9,19^

### Novel long-lived deletion mutants indicate unexplored mechanisms of aging

**Fig. 3:**
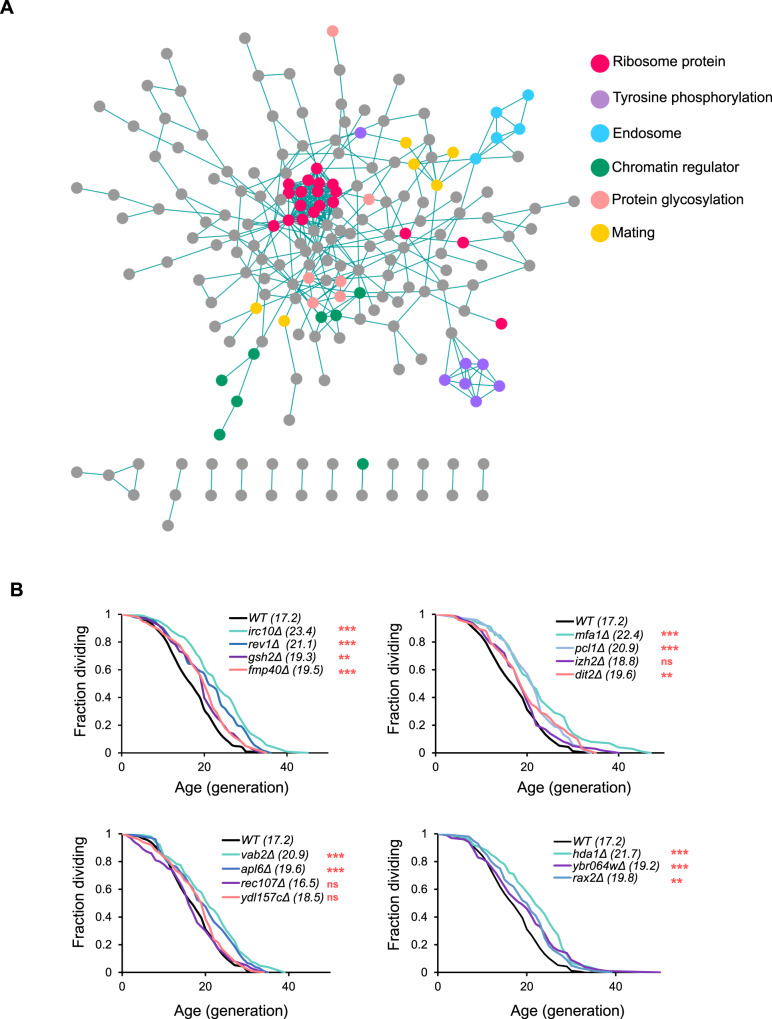
Gene interaction network analysis and microfluidic validation of SEBYL results. **A** Functional clustering of 285 long-lived deletion mutants. Circles represent long-lived deletions; edges are published physical protein–protein interactions. Overrepresented categories are highlighted in designated colors. **B** RLS of designated strains of both a and alpha mating types combined. Average RLS is noted in parenthesis. *p*-value calculated by two-sided Mann–Whitney *U* test. ns not significant, **p < 0.01, ***p < 0.001. See Supplementary Data 3 for all statistical analysis on RLS experiments. See also Fig. S2.

We identified 285 putative long-lived and 288 putative short-lived mutants (one standard deviation above and below the mean, see Supplementary Data 2 for complete results of SEBYL screen). The long-lived candidates clustered into several functional groups, including ribosome proteins, tyrosine phosphatase, glycosylation genes, endosome, mating, and chromatin regulatory genes. Ribosome proteins and chromatin regulators have well-documented roles in aging, and also appeared as clusters in a published manual microdissection screen (Fig. 3A)^6^. We also discovered a less-characterized cluster enriched with tyrosine phosphorylation regulators in our network analysis. This cluster included six tyrosine phosphatase genes, *PTP2*, *SIW14*, *OCA1*, *OCA2*, *OCA4*, and *OCA6*. Of these phosphatases, Ptp2 is known to target the MAP kinase Hog1, a major stress regulator in budding yeast, and also play a role in osmolarity stress response^23,24^. The molecular functions of the other candidate phosphatases are less well characterized, but all of them except *OCA6* have extended chronological lifespans, according to a previous study^24^, suggesting loss of function in these tyrosine phosphatases may lead to enhanced resistance against external stress, which tends to extend chronological lifespan^25^.

To further identify and validate the long-lived deletion mutants, we chose 15 long-lived deletion strains and performed a microfluidics-based RLS measurement. These strains had no conclusive RLS data from the manual screen and ranked among the highest in our candidate list. We confirmed that 12 out of the 15 strains were significantly longer-lived than wild type (Fig. 3B), with similar results from microdissection experiments (Fig. S2A), further supporting the reliability of our screening method. It should also be noted that these genes represent several pathways that are poorly understood in the context of aging regulation. Among these pathways, *MFA1* and *DIT2* are involved in sexual reproduction; *PCL1* and *RAX1* are regulators of cytokinesis. We believe future studies of these long-lived candidates could reveal previously unknown molecular mechanisms of aging.

### Mutations in the HDA complex lead to longevity

**Fig. 4:**
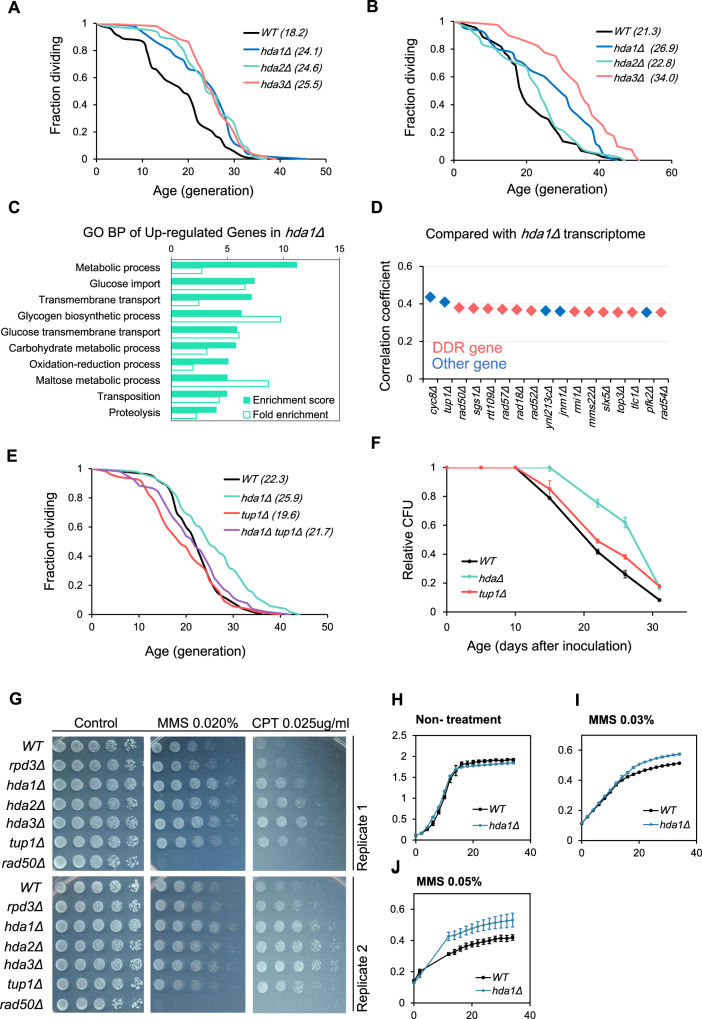
HDA complex mutants have extended lifespan and enhanced genotoxic resistance. **A** RLS of WT, *hda1Δ*, *hda2Δ*, and *hda3Δ* measured by microfluidic method. **B** RLS of WT, *hda1Δ*, *hda2Δ*, and *hda3Δ* measured by manual microdissection. **C** Gene ontology biology process analysis on upregulated genes on *hda1Δ* transcriptome. Pathways with Benjamini *p*-value > 0.05 were selected. **D** Deletion strains of which transcriptomes have highest correlation coefficient with *hda1Δ*. Genes involved in DNA damage response (DDR) is labeled in red. **E** RLS of WT, *hda1Δ*, *tup1Δ*, and *hda1Δ tup1Δ* measured by microfluidic method. **F** Chronological lifespan (CLS) of WT, *hda1Δ* and *tup1Δ*. Error bars represent standard error (SEM) calculated from three biological replicates. **G** Drug sensitivity test on designated conditions. Control plate used here is synthetic complete (SC) agar plate. **H** Growth curve of WT and *hda1Δ* in synthetic complete (SC) media, over period of 34 h. Error bars represent SEM, *n* = 3. **I** Growth curve of WT and *hda1Δ* in synthetic complete (SC) media, with 0.03% MMS, over period of 34 h. Error bars represent SEM, *n* = 3. **J** Growth curve of WT and *hda1Δ* in synthetic complete (SC) media, with 0.05% MMS, over period of 34 h. Error bars represent SEM, *n* = 3.

Among candidate mutants with the greatest lifespan extensions, we found deletion strains of two subunits of histone deacetylase complex HDA: *hda1Δ* and *hda2Δ*. There are four classes of histone lysine deacetylases: Class I, II, III, and IV, which are classified by sequence similarity^26^. HDA is a Class-II histone deacetylase complex made up of three subunits: the catalytic subunit Hda1 and accessory factors Hda2 and Hda3^27^. HDA complex specifically deacetylates acetylated lysine on H3, but has less activity on histone H4^28^. Curiously, a previous study found no significant lifespan change in an *hda1Δ* mutant^29^. However, SEBYL results, coupled with microfluidic and microscopic dissection validation, suggested that mutations of all three HDA components would lead to robust lifespan extension (Fig. 4A, B).

To reveal the mechanism behind *hda1Δ* mediated RLS extension, we analyzed the transcriptome by RNA-sequencing (RNA-seq). GO analysis showed significant upregulation of several carbohydrate metabolism related pathways, such as glucose import, glycogen metabolism, etc. (Fig. 4C, see Supplementary Data 4 for complete list). To better understand the molecular process represented by these changes, we compared the *hda1Δ* transcriptome to a previously published dataset containing the transcriptome of 1400 single gene deletion strains^30^, to find mutants with highly similar transcriptomes as that of *hda1Δ*. The most similar mutants we identified were *tup1Δ*, *cyc8Δ*, and several factors involved in DNA damage repair (DDR), such as homologous recombination factor Rad50 and DNA helicase Sgs1 (Fig. 4D).

Tup1 and Cyc8 form the Tup1/Cyc8 transcription repression complex in budding yeast. The complex represses expression of several stress response genes, including genes that regulate glucose and oxygen usage, DNA damage, osmotic stress, and other signals^31^. Previous studies showed that the Tup1/Cyc8 complex recruits Hda1, and another class I histone deacetylase, Rpd3, to target genes, inducing promoter deacetylation and transcription inactivation, explaining the transcriptomic similarity between *hda1Δ* and *tup1Δ/cyc8Δ* strains^27^. Additionally, it was reported that *rpd3Δ* could also extend lifespan^29^, similar to *hda1Δ*. To test whether the longevity phenotype of *hda1Δ* was indeed mediated by Tup1/Cyc8-dependent gene inactivation, we used epistasis analysis to determine whether *hda1Δ* mutation could still extend RLS in a *TUP1* knockout background. In contrast to this prediction, we observed that *TUP1* deletion did not extend lifespan on its own, but actually slightly shortened RLS, and deletion of *HDA1* extended lifespan even in cells lacking *TUP1* (Fig. 4E). These results indicate that the effect of *hda1Δ* on lifespan is likely independent of *TUP1* function. Apart from replicative lifespan (RLS), chronological lifespan (CLS) defines the time yeast are able to survive after nutrient depletion and cell cycle cessation, which is believed to be tightly connected with the stress resistance ability of cells^32^. Since Tup1/Cyc8 complex is a known repressor of stress response, we tested whether the CLS of *tup1Δ* and *hda1Δ* mutants showed any difference. We confirmed that only *hda1Δ* extended CLS, further supporting the conclusion that *TUP1* deletion has little consequence on lifespan outcome (Fig. 4F).

Ruling out Tup1/Cyc8, we shifted our focus to DDR pathways, because DDR mutants have highly similar transcriptomes to that of *hda1Δ*. Inhibition of histone deacetylase activity by HDAC inhibitors is reported to hinder the DNA damage response^33^; thus, it is possible that *hda1Δ* leads to a defective DDR, causing genotoxic stress and activating downstream stress response genes. Another possibility is that *HDA1* deletion does not affect DDR directly, but upregulates downstream adaptive responses activated by genotoxic environments. To narrow the field of possibilities, we used a growth assay to ask how HDA mutation affects cellular resistance to DNA damaging chemicals. The growth assay showed that all three HDA mutant strains had increased resistance to the genotoxic drugs methyl methanesulfonate (MMS) and camptothecin (CPT; Fig. 4G), similar increase of MMS resistance in *hda1Δ* was also seen in study conducted by another group^34^. Additionally, we confirmed that growth of *hda1Δ* is less inhibited in MMS containing liquid media compared to wild type by measurement of growth curve (Fig. 4H–J). This result strongly favors the second hypothesis that *HDA1* deletion upregulates downstream adaptive responses. Additionally, we found that the stress resistance of the *rpd3Δ* strain was not nearly as strong as in *HDA* mutants. Although a lifespan extension effect from *rpd3Δ* is previously reported^29^, this finding was not repeatable in our own experiments and other published results using the BY4741 background^2^. These findings are evidence that indicate potential different functions of HDA and Rpd3 in lifespan regulation.

### HDA- dependent longevity is mediated via trehalose metabolism

**Fig. 5:**
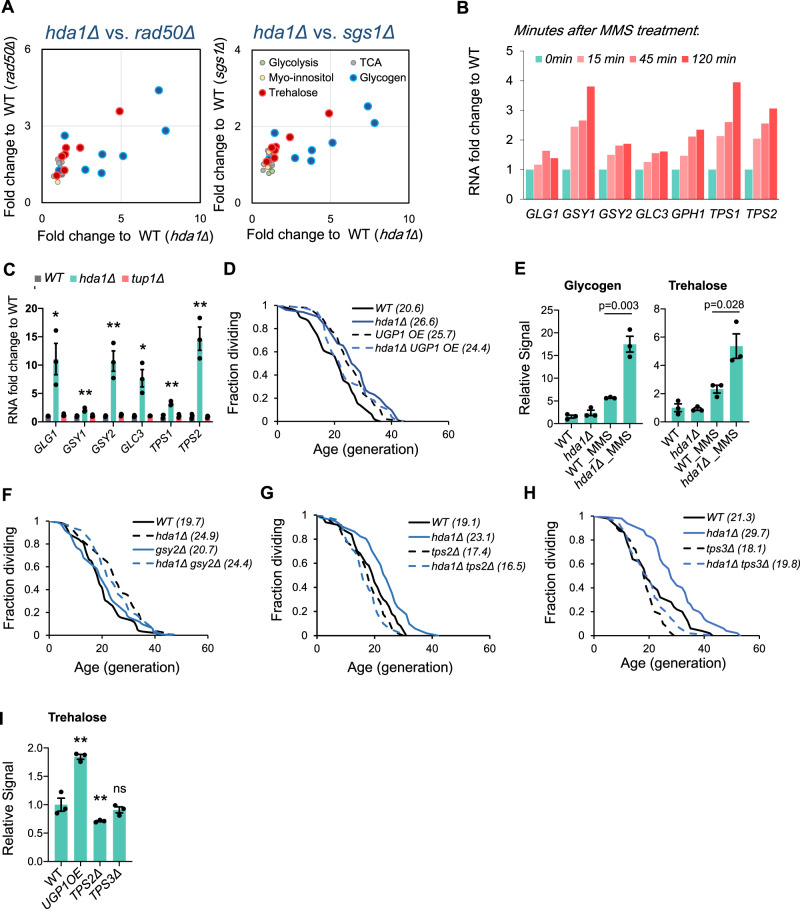
Longevity of HDA mutants is dependent on trehalose metabolism. **A** Transcriptome analysis of five carbohydrate metabolism related pathways in *hda1Δ, rad50Δ*, and *sgs1Δ*. **B** RNA level of designated glycogen and trehalose metabolism genes in *hda1Δ* and *tup1Δ*, data from Reimand et al.^45^. **C** RNA level of designated glycogen and trehalose metabolism genes in *hda1Δ* and *tup1Δ*, measured by quantitative real-time PCR. Error bars represent SEM, *n* = 3. *p*-value calculated by two-sided T-test, *p < 0.05, **p < 0.01. Exact *p*-values are *GLG1*: 0.022; *GSY1*: 0.007; *GSY2*: 0.006; *GLC3*: 0.012; *TPS1*: 0.002; *TPS2*: 0.003. **D** RLS of WT, *hda1Δ*, *UGP1OE*, and *hda1Δ UGP1OE*. **E** Glycogen and trehalose levels in WT and *hda1Δ*, with designated time of treatment under MMS. Error bars represent SEM, *n* = 3. **F** RLS of WT, *hda1Δ*, *gsy2Δ*, and *hda1Δ gsy2Δ*. **G** RLS of WT, *hda1Δ*, *tps2Δ*, and *hda1Δ tps2Δ*. **H** RLS of WT, *hda1Δ*, *tps3Δ*, and *hda1Δ tps3Δ*. **I** Trehalose levels in WT, *UGP1OE*, *tps2Δ* and *tps3Δ*. Error bars represent SEM, *n* = 3. Exact *p*-values are *UGP1OE*: 0.002; *tps2Δ*: 0.002 and *tps3Δ*: 0.50. See also Fig. S3.

We found no change in DDR pathways genes by gene ontology analysis (Fig. 4C), bringing up the question of how disrupting the HDA complex can contribute to enhancement of cellular resistance to genotoxic stress. As carbohydrate metabolism genes were significantly upregulated in *hda1Δ*, we aimed to identify the specific pathway that was involved. We analyzed five metabolic pathways that involved carbohydrates as substrates or products: glycolysis, tricarboxylic acid (TCA) cycle, myo-inositol metabolism, glycogen metabolism, and trehalose metabolism. We found that only glycogen and trehalose pathways were significantly upregulated in both *hda1Δ* and two DDR mutant strains: *rad50Δ* and *sgs1Δ* (Fig. 5A). Both glycogen and trehalose are conserved storage carbohydrates; glycogen plays a vital energy storage role conserved from budding yeast to humans, whereas trehalose also serves as storage carbohydrate in other fungi, plants, nematodes, and insects, although it is not synthesized in mammals. Notably, trehalose has long been known to protect cells from extracellular stress: in budding yeast, trehalose protects against desiccation, high temperature, freezing, high ethanol concentration, and osmotic and oxidative stresses^35–38^, and was recently proposed to play a vital function in adaptation to stress by maintaining viscosity homeostasis^39^. Trehalose has a similar protective role in the nematode *C. elegans*^40^, and its metabolism is essential for survival of the fruit fly *D. melanogaster*^41^. Although trehalose is not synthesized in mammals, mouse consumption of trehalose increases longevity in some contexts, by enhancing autophagy in neuronal tissue^42^. Neither glycogen nor trehalose are reported to function in the response to DNA damage, but levels of both acutely increase upon genotoxic stress^43^. Using a published dataset^44^, we confirmed that all glycogen and trehalose metabolism genes were upregulated upon MMS treatment (Fig. 5B), confirming that metabolism of these two carbohydrates responds to genotoxic stress. Furthermore, we used real-time PCR to verify that glycogen and trehalose synthesis enzymes were significantly upregulated in *hda1Δ*, but not *tup1Δ* mutants (Fig. 5C). Previous RNA -seq data also support this conclusion (Fig. S3A)^45^. Uridine diphosphate glucose (UDP- glucose) is a substrate for both trehalose and glycogen metabolism^46^. We found that overexpression of the UDP-glucose pyrophosphorylase gene *UGP1* extended yeast lifespan, and this extension was epistatic with *hda1Δ* (Fig. 5D), suggesting an active role for glycogen and/or trehalose metabolism in mediating the *hda1Δ* longevity phenotype.

Because synthases and hydrolases were both upregulated in an *hda1Δ* mutant, it is unclear how intracellular glycogen and trehalose levels respond to *HDA* gene deletion. We measured glycogen and trehalose levels with established methods^47^ and confirmed that, although levels of neither carbohydrates were significantly different under normal growth condition, both glycogen and trehalose levels were drastically increased in *hda1Δ* mutant under treatment with MMS (Fig. 5E). This result strongly suggests that the accumulation of either one or both carbohydrates contributes to the enhanced genotoxic resistance of HDA mutants. To further determine if one of these two molecules plays more important role in stress resistance, we tested whether trehalose or glycogen was involved in genotoxicity resistance in *HDA* mutants. We found that deletion of glycogen synthase *GSY2* had no epistatic effect on *hda1Δ* mutants *(*Fig. 5F). In contrast, knockout of *TPS2* or *TPS3*, the phosphatase and regulatory subunit of trehalose synthase respectively, completely suppressed the longevity phenotype of *hda1Δ* (Fig. 5G, H). We also found that the long-lived *UGP1OE* strain to have significantly upregulated trehalose level, while the trehalose level is decreased significantly in *TPS2* mutant (Fig. 5I). Furthermore, we found that *hda1Δ* cells also have higher resistance to high concentration of sodium chloride (Figure S3B), consistent with the fact that trehalose protects cells from osmotic stress^48^. These findings led us to conclude that disruption of HDA function increases trehalose gene expression and its level under stress, and that extended lifespan in *hda1Δ* cells was mediated by trehalose metabolism, not glycogen.

### Mutation of Class-II histone deacetylases shows conserved lifespan phenotypes

**Fig. 6:**
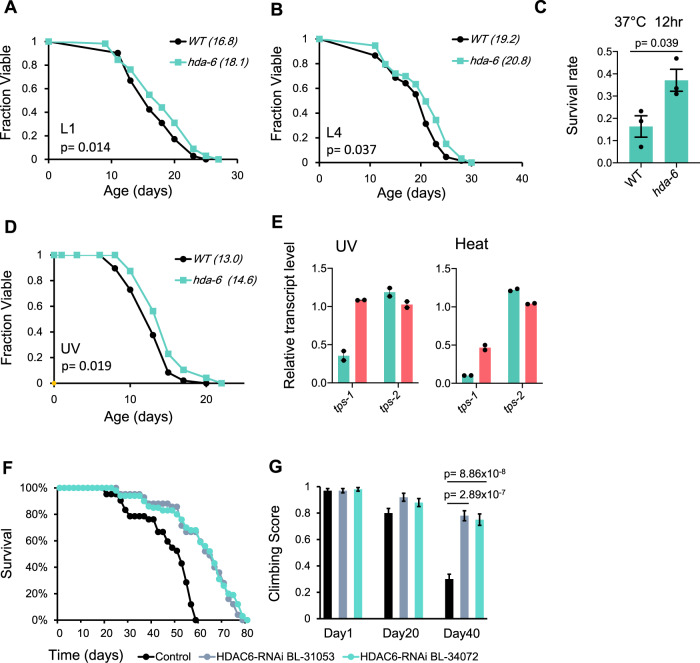
Evolutionary conservation of HDA function. **A** Lifespan of WT and *hda-6* knockdown worms, RNAi treatment started at L1 stage, *n* = 176 for WT and 169 for *hda-6*. Statistical significance was calculated by two-sided Mann–Whitney *U* test. **B** Lifespan of WT and *hda-6* knockdown worms, RNAi treatment started at L4 stage. *n* = 67 for WT and 93 for *hda-6*. Statistical significance was calculated by two-sided Mann–Whitney *U* test. **C** Survival rate of WT and *hda-6* knockdown worms after 12 h incubation in 37 °C. Error bars represent SEM, three independent experiments were performed with 32 worms in each group. Statistical significance was calculated by two-sided *t*-test. **D** Lifespan of WT and *hda-6* knockdown worms under 0.08 J/cm^2^ UV treatment. *n* = 50 for control and UV treatment group each. Statistical significance was calculated by two-sided Mann–Whitney *U* test. **E** RNA level of *tps-1* and *tps-2* genes in WT and *hda-6* KO worms, under UV and heat. Two biological replicates were included in each group. **F** Lifespan analysis at 25 °C. *N* = 300 flies. Control group genotype: tub-Gal4, tub-Gal80ts / CyO; HDAC6 silencing group genotype: tub-Gal4, tub-Gal80ts/UAS-HDAC6-RNAi. Two independent *HDAC6* RNAi lines (BL-31053 and BL-34072) were tested to avoid potential off-target effect. About 100 flies were tested for each group. **G** Climbing ability analysis at 5-, 20-, and 40-day-old adult flies. In all, 40 adult flies were tested in each age/genotype group. The results were presented as mean ± SEM. Statistical significance was calculated by two-sided Mann–Whitney *U* test.

Class-II histone deacetylases are conserved from yeast to humans. In mammals, there are six members of Class-II HDAC: HDAC4, 5, 6, 7, 9, and 10, and their roles in neuronal physiology and immunity were identified in mice^49^. However, evidence for Class-II HDAC activity in aging is still lacking. As such, we asked whether the effect of HDA mutation was conserved during evolution. Because mammals do not synthesize trehalose, we tested whether Class-II HDAC mutants had similar lifespan phenotypes in the nematode *Caenorhabditis elegans* and the insect *Drosophila melanogaster*. *C. elegans* has 5 Class-II HDACs: *hda-4, hda-5, hda-6, hda-10*, and *hda-11*^50^. We found that one of these genes, *hda-6*, significantly extended lifespan when knocked-down by RNAi at both L1 and L4 developmental stages (Fig. 6A, B). Since Hda1 regulates stress resistance in yeast, we next tested whether *hda-6* deficiency could result in higher stress resistance in worms as well. Indeed, we confirmed that worms with *hda-6* knockdown were more resistant to heat and UV treatment (Fig. 6C–D). Moreover, we found that one of the trehalose synthase gene in worm, *tps-1*, was activated to a higher level in the *hda-6* KO strain under UV and heat treatments (Fig. 6E).

In order to test whether silencing of *HDAC6*, the *Drosophila* homolog of yeast *HDA1*, could increase adult fly lifespan, we used a ubiquitous driver tubulin-Gal4 (tub-Gal4) to knockdown the expression of *HDAC6* in all the fly cells. However, the embryos died before hatching to 1st instar larvae, suggesting that *HDAC6* is required for early embryo development in *Drosophila* (Supplementary table S1). To overcome this problem and test the effects of *HDAC6* knockdown in aging adult flies, we applied the Gal80 system with tub-Gal4, tub-Gal80ts. This method allows us to keep the expression of *HDAC6* unchanged during embryo, larvae and pupa stages by keeping the flies in 18 °C, and then knockdown *HDAC6* expression after adult fly emergence by keeping newly hatched adult flies in 25 °C, at this temperature the Gal80ts allele lost its inhibition of Gal4, allowing the expression of *HDAC6* RNAi to silence the expression of *HDAC6* in adult flies.

We found that *HDAC6* silencing in adult flies increased the adult lifespan by almost 25% (Fig. 6F). In the control group without *HDAC6* RNAi, half of the adult flies died at day 50 and all of them died before day 60. With the *HDAC6* silencing in adult flies, more than 60% flies are alive at day 60. Half of the adult flies died at day 65, and all of them died around day 80. We also compared the climbing ability in different aged flies (Fig. 6G). At day 5, there was no difference between control and *HDAC6* silencing flies. At day 40, the climbing ability in control flies dramatically reduced, while *HDAC6* silencing flies still have normal physical strength, suggesting that silencing of *HDAC6* in adult flies not only expands lifespan, but also delays aging-associated physical function decline, at least in *Drosophila*. In conclusion, these results suggest that the previously uncharacterized functions of Class-II HDACs in aging regulation may be evolutionarily conserved.

### The lifespan effect of *hda1Δ* is mediated by H3K18 deacetylation

**Fig. 7:**
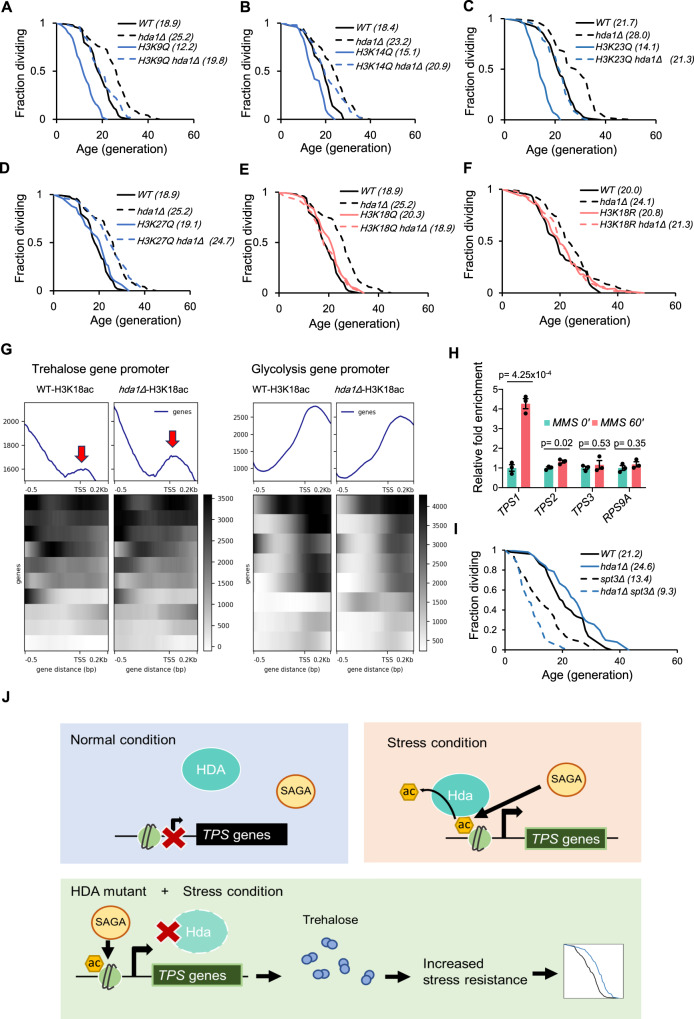
The lifespan phenotypes of HDA mutants are mediated by H3K18 acetylation. **A** RLS of WT, *hda1Δ*, *H3K9Q*, and *hda1Δ H3K9Q*. **B** RLS of WT, *hda1Δ*, *H3K14Q*, and *hda1Δ H3K14Q*. **C** RLS of WT, *hda1Δ*, *H3K23Q*, and *hda1Δ H3K23Q*. **D** RLS of WT, *hda1Δ*, *H3K27Q*, and *hda1Δ H3K27Q*. **E** RLS of WT, *hda1Δ*, *H3K18Q*, and *hda1Δ H3K18Q*. **F** RLS of WT, *hda1Δ*, *H3K18R*, and *hda1Δ H3K18R*. **G** Metagene plot and heatmap of H3K18 acetylation on trehalose (left) and glycolysis (right) promoters as detected by ChIP- Seq. **H** ChIP-qPCR of Hda1-myc binding on *TPS1, TPS2, TPS3*, and *ACT1* promoters, under designated time after MMS treatment. Error bars represent SEM, *n* = 3. *p*-value calculated by two-sided T- test. **I** RLS of WT, *hda1Δ*, *spt3Δ*, and *hda1Δ spt3Δ*. **J** Mechanism of HDA mediated suppression of trehalose metabolism and its effect on yeast aging. See also Figure S4.

As a protein deacetylase, HDA regulates cellular activity by deacetylating histones and changing gene transcription activity, or by deacetylating non-histone proteins that regulate stress responses. The HDA complex specifically targets histone H3 lysine acetylation with little activity on H4^28^, so we asked which lysine residue on H3, if any, was responsible for the lifespan phenotype caused by *hda1Δ*. We constructed double mutants by crossing *hda1Δ* mutants with H3 lysine point mutants that were constructed by Dai et al.^51^, and found no epistasis effects in H3K9, K14, K23, or K27 mutants (Fig. 7A–D). However, mutation of the H3K18 residue, regardless of whether it was lysine to glutamine (Q) or lysine to arginine (R), showed an epistatic relationship with *hda1Δ* (Fig. 7E, F). We also confirmed that deletion of *HDA1* results in apparent increase in H3K18 acetylation level (Fig. S4A). Previous report showed that *HDA1* deletion significantly increases H3K18 acetylation at promoters of the *ENA1* gene^27^, which is regulated by the *TUP1/CYC8* complex through HDA mediated deacetylation. We hypothesize that a similar increase of H3K18 acetylation at promoters could occur at trehalose metabolism genes with *HDA1* deletion and contribute to enhanced transcription of these genes. Using ChIP-seq to detect H3K18 acetylation, we confirmed an increase in promoter acetylation at trehalose metabolism genes (Fig. 7G). These changes were not visible on genes not activated by *HDA1* deletion, such as glycolysis pathway genes, suggesting that HDA mediated histone deacetylation is selective. Gene ontology analysis showed a significant increase in H3K18 acetylation at promoters of genes in stress response, drug response, and pentose transport categories (Fig. S4B), which is consistent with the increased stress resistance and upregulation of carbohydrate transportation we observed (Fig. 4C, G). Furthermore, these H3K18 acetylation increases in *hda1∆* correlate well with elevated expression of these genes (Fig. S4C), confirming that HDA regulates the expression of target genes through H3K18 acetylation.

As a histone deacetylase, the HDA complex is expected to physically interact with gene promoters and deacetylate histones. Indeed, there is demonstrated physical interaction between the HDA complex and target promoters^52^. Since it is a transcriptional repressor for stress resistance genes, we asked whether genotoxic stress could remove the HDA complex as part of stress adaptation. Surprisingly, Myc-tagged Hda1 protein recruitment to *TPS1* and *TPS2* promoters increased under MMS treatment, whereas there was no significant change at the control gene *RPS9A* promoter (Fig. 7H). Thus, the level of HDA recruitment under genotoxic stress positively correlated with trehalose gene expression levels, which is consistent with the fact that removal of the HDA complex enhances genotoxic resistance. Since histone deacetylases have higher affinity to acetylated histones than unacetylated ones, it is likely that increased recruitment of the HDA complex during genotoxic stress is caused by increased recruitment of histone acetyltransferases and hence histone acetylation levels.

During stress conditions, stress response genes are acetylated by the SAGA complex in budding yeast^53^. SAGA is an evolutionarily conserved histone acetyltransferase complex that acetylates K9, K14, K18, K23, and K27 on histone H3^54^. Using data from a previous study^55^ we found that MMS treatment enriched SAGA component Spt3 at promoter regions of trehalose genes (Fig. S4D). We also confirmed that the lifespan extension phenotype of *hda1Δ* depends on a functional *SPT3* gene (Fig. 7I). These findings suggest that genotoxic conditions cause the SAGA complex to be increasingly recruited to trehalose gene promoters and creates the acetylated chromatin that facilitates HDA complex recruitment.

## Discussion

In this study, we developed a barcode sequencing-based technique, SEBYL, to perform high-throughput screening for yeast mutants with an altered RLS. We demonstrated that SEBYL yielded results that are consistent with classical experiments, yet taking <1 month to perform. Our screen also improved on data for strains with previously inconclusive RLS data that had been generated by classical screening and identified several long-lived candidates not previously reported. We conclude SEBYL to be fast, reliable, and suitable for RLS screens of many different barcoded yeast mutant collections.

Using SEBYL, we identified a previously unknown regulator of aging, the histone deacetylase complex HDA. We showed that strains with defective HDA function were more resistant to various stressors, especially genotoxicity. This resistance depended on the metabolism of storage carbohydrate trehalose, of which metabolism genes were significantly upregulated in *hda1Δ* cells, potentially protect yeast cells from genotoxic stress. We also demonstrated that genotoxic stress caused the SAGA complex to bind the trehalose promoter. This binding likely induced histone acetylation, which in turn mediated HDA recruitment to target locations. Based on these results, we propose the following model: Under genotoxic stress, the HDA complex is recruited to trehalose promoters by acetylated histones, which inhibits expression of trehalose genes via promoter deacetylation. When HDA is non-functional, the acetylation at promoters of trehalose genes increases and activates expression of trehalose genes. Increased trehalose gene expression promotes stress resistance, thus extending the lifespan of yeast cells (Fig. 7J).

### SEBYL is a fast and reliable approach to RLS screening in budding yeast

Microdissection and microfluidic-based RLS measurements are not truly high-throughput, due to the requirement for manual cell counting. In both methods, determining cell division events requires judgement by eyesight, which is time and energy consuming. Compared to previously published yeast RLS screening approaches, the SEBYL method has the following advantages: (1) A considerably faster process where all benchwork can be finished in only 2 weeks; (2) SEBYL does not require additional instruments, such as an automated flow cytometer. Only the reagents for old cell sorting and next-generation sequencing access are needed; (3) Because SEBYL uses the entire pool of yeast collection, results are based on an unbiased screen of all mutants present in one collection; (4) SEBYL does not require strains with a specific genetic background. All barcoded yeast collections can be used; (5) SEBYL is demonstrably reproducible and generates lifespan data comparable to results obtained from both microdissection and microfluidic methods. Here, we demonstrate the use of SEBYL on yeast gene knockout collection and proved it to be a valid way to complement existing methodologies of RLS screening.

Apart from identifying genes involved in aging regulation, SEBYL also generates large datasets for bioinformatic analysis. Bioinformatic studies using large-scale datasets are increasingly important for characterizing gene functions and molecular pathways^56^. Here we demonstrated that SEBYL screening data could identify cell morphology traits associated with both longer and shorter lifespans. Due to technical difficulties of large-scale lifespan screening, there is still a lack of comprehensive RLS data collections. We anticipate that the development of SEBYL will facilitate generation of RLS datasets and provide valuable resources for bioinformatic studies of aging biology.

### HDA regulates aging through trehalose metabolism independent of Tup1/Cyc8

The Tup1/Cyc8 complex is transcriptional corepressor complex with functional similarity to metazoan corepressor protein such as Groucho protein in Drosophila, and Transducin-like Enhancer of Split (TLE) family in mammalian cells^57^. Previous study shows Tup1/Cyc8 complex acts as an inhibitor of various stress resistance genes by recruiting histone deacetylase Rpd3 and HDA complex on target promoters^27^. In this study, we provide evidence that inactivation of HDA complex could lead to increased stress resistance and lifespan extension independent of Tup1/Cyc8 function: First of all, removal of *TUP1* gene was not able to extend replicative lifespan, nor was it able to suppress RLS extension brought by *hda1Δ* (Fig. 4E); Secondly, *tup1Δ* and *rpd3Δ* strains did not show significantly enhanced genotoxic resistance as seen in three *HDA* mutants (Fig. 4G). Furthermore, the significant induction of trehalose metabolism genes was also not recapitulated in *tup1Δ* mutant (Fig. 5C and D).

These lines of evidence suggest that just like class I histone deacetylase Rpd3^58^, HDA may also have distinct target profiles depending on different enzymatic complexes it resides in. In this study, we identified the trehalose metabolism genes to be especially important targets of HDA complex mediated deacetylation. Trehalose levels increase drastically under stress, which has been proposed to be an important protectant to organisms^33–36^. We found that abrogating HDA releases the inhibition of trehalose metabolism genes (Fig. 7G), and therefore leads to higher resistance to various source of stress (Figs. 4G and S2B). Though we are not able to decipher how increased trehalose benefits stress response, a recent study^39^ shows that trehalose is essential for maintaining cellular viscosity homeostasis, making cells adapt better to different environmental conditions. Further study on detailed mechanisms behind the protective functions of trehalose may lead to novel intervention strategies for aging and age-related diseases.

### Class-II histone deacetylases may have additional roles in aging in higher eukaryotes

This study contains the first direct evidence to suggest that deletion of Class-II deacetylase HDA extends lifespan, such effect is also conserved from unicellular budding yeast to metazoan *C. elegans* and *D. melanogaster*. We determined that at least in budding yeast, the effect of *hda1Δ* on lifespan is mediated through released inhibition of trehalose metabolism genes, which leads to enhanced stress resistance and extended lifespan.

Post-translational modification (PTM) of histones changes significantly during aging, and is characterized by a general opening of chromatin structure; histone PTMs are thus proposed to play important roles in aging regulation^59^. Histone acetylation and deacetylation are vital to transcriptional regulation, but these processes are also heavily implicated in cellular senescence and organismal aging^60^. Histone deacetylases (HDACs) are extensively studied with regard to their functions in the aging process: Sirtuins, one of the most studied groups of anti-aging factors, are nicotinamide adenine dinucleotide dependent Class-III lysine deacetylases. The anti-aging activity of the sirtuin protein family is evolutionarily conserved from yeast to mouse and regulates various pathways, including gene silencing, nutrition sensing, and stress response^61^. Class I deacetylases, such as yeast Rpd3 and human HDAC1, are also proposed to affect aging by various mechanisms^62,63^.

Unlike class I HDACs and sirtuins, the function of Class-II HDACs in aging is relatively unknown and the existing data is sometimes contradictory. For example, the reduction of HDAC4 is shown to facilitate clearance of pathogenic protein that aggregates in a Huntington disease model and antagonizes neuron degeneration^64^. In contrast, another study reported HDAC4 overexpression delays cellular senescence via SIRT1 sumoylation^65^. Because we observed increased lifespan phenotypes after ablating *HDA* in multiple model organisms, it will be interesting to see whether inhibiting any of the Class-II HDACs affects lifespan in mammals. Additionally, the relationship between Class-II HDACs, trehalose metabolism and aging still needs to be explored in greater detail. Although we provided evidence supporting that Hda1 regulates aging through trehalose metabolism in the budding yeast, it remains to be seen whether such mechanism stays similar throughout evolution. In conclusion, we believe Class-II HDACs play important roles in aging regulation, and hence could be feasible targets to combat age-related diseases because multiple known HDAC inhibitors are already used in treating cancer^66^.

## Methods

### Yeast deletion strain pool construction and old cell isolation

Yeast library was pinned to YPD plates and grown for 3 days, then scrapped into 50 ml centrifuge tube with YPD and 200 μg/ ml G418 (Thermo Fisher Scientific)^67^. Additional three barcoded wild-type BY4742 strains were pooled together. Biotin-based old cell isolation method^4^ was used to isolate young and old cells for analysis. Briefly, cells were harvested at OD600~1.0, labeled with EZ-link Biotin (Thermo Fisher Scientific) at a concentration of 1 mg/10^8^ cells. After wash for three times with PBS containing 0.1 M glycine, cells were resuspended in synthetic complete (SC) medium and incubated overnight. The cells were then incubated with Dynabeads Biotin Binder (Thermo Fisher Scientific) at 4° for 1 h. Cells are then washed with PBS, with first wash kept as young cell control. Isolated mother cells were inoculated in SC media again, the process was repeated four times to get four samples with increasing replicative age.

### High-throughput lifespan screen with the yeast deletion library

The pool of mutants was cultured and subjected to four successive rounds of old mother sorting to save ~10^8^ old cells after sorts 1, 2, 3, and 4 (S1O, S2O, S3O, and S4O). Equivalent number of young cells after each round of sorting (S1Y, S2Y, S3Y, and S4Y) as well as an aliquot prior to aging and sorting (USC) were also saved. Genomic DNA was extracted from young and old fractions using standard methods and the barcodes UPTAG and DNTAG were quantified by next-generation sequencing^15^. To prepare next-generation sequencing libraries, primers U1d-F1(see table S2. For all primers used in this study) and U2d-R1 were used to amplify UPTAG, and primers D1d-F1and D2d-R1 were used for DNTAG. All next-generation sequencing libraries were indexed and sequenced in Illumina HiSeq 2500. All data were converted to fold change compare with USC and designated as relative enrichment.

### Replicative lifespan assay by microfluid

Microfluidic-based yeast RLS measurement was performed with Invitrogen EVOS FL Auto Imaging System^68^. Briefly, cells were grown overnight in filter-sterilized yeast extract, peptone, and dextrose (YPD) medium, diluted 20-fold with fresh medium, and loaded onto a microfluidic chip. Medium flow speed was set at 10 μl/min, and pictures were taken at 10-min intervals for 65 h. All experimental data and statistical analysis are provided in Supplementary Data 3.

### Replicative lifespan assay by microdissection

Microdissection measurement of replicative lifespan is based on existing protocol as described in McCormick et al.^6^. Virgin daughter cells were collected and plated, allowed to grow into mother cells. The daughter cells were micro-dissected and counted until mother cells cease dividing.

### RNA extraction, RNA-seq library preparation, and next-generation sequencing

Yeast cells were lysed in QIAzol buffer (QIAGEN) with 0.5-mm zirconia/silica beads (BioSpec) for four cycles. Poly-A RNA was purified from 5 μg total RNA with Dynabeads Oligo (dT)25 (Thermo Fisher Scientific). Sequencing libraries were prepared with NEBNext Ultra Directional RNA Library Prep Kit for Illumina (New England BioLabs). Sequencing of three biological replicates was performed using the Illumina HiSeq 2500 platform.

### Chronological lifespan measurement

Yeast cells were inoculated in YPD and incubated for 3 days as starting culture, with three replicates for each strain. At designated time point (every 5 or 6 days apart), a sample of yeast culture were collected and diluted 10^6^ folds, then spread onto YPD plates. Number of colonies were counted and relative colony-forming units (CFU) compared to day 0 culture were calculated.

### Yeast growth assay

Yeast cells were grown overnight at 30 °C, diluted to OD600 of 0.1, then potted in fivefold serial dilutions on designated agar plates. Control plates (SC) were incubated at 30° for 24 h and all other drug plates for 48 h. Plates were scanned with Epson Perfection V500 Photo scanner.

### RNA extraction, reverse transcription PCR, and real-time quantitative PCR analysis

Yeast cells with total OD600 of 1 were lysed in QIAzol buffer (QIAGEN) with 0.5-mm zirconia/ silica beads (BioSpec) for four cycles. Total RNA was purified with miRNeasy Mini Kit (QIAGEN). Reverse transcription was performed with the High-Capacity cDNA Reverse Transcription Kit (Thermo Fisher Scientific) using 1 μg of purified RNA. Real-time PCR was performed with ViiA 7 Real-Time PCR System (Thermo Fisher Scientific) and data analysis performed by QuantStudio Real-Time PCR software. Yeast gene expression data were normalized to the *ACT1* or an intragenic negative control (iYHL004W). All qPCR primers used are listed in Table S6.

### Trehalose and glycogen measurement

Measurement of glycogen and trehalose were performed according to published protocol^47^. Briefly, ~10^8^ cells were harvested and washed with 1 ml ice-cold water. Cells were resuspended in 125 μl of 0.25 M Na_2_CO_3_ and incubated at 95 °C for 3 h. pH was adjusted to 5.5 with 75 μl 1 M acetic acid and 300 μl 0.2 M NaAcetate, pH 5.2 then separated into two tubes for glycogen and trehalose measurement. For glycogen, 20 mg/ml solution of Aspergillus niger α-amyloglucosidase (Sigma) was added and incubated at 57 °C overnight. For trehalose, 15 μl of 0.2 M NaAcetate and 3 μl of porcine trehalase (Sigma) was added and incubated at 37 °C overnight. Released glucose was measured with Glucose assay kit (Sigma-Aldrich) and read by BioTek Synergy 2 Multi-Mode Reader, with the data analyzed using the Gen5 software.

### Protein extraction and quantitative western blot analysis

Yeast cells were harvested at OD600 of 0.8–1.0 by centrifugation at 3000 × *g* for 3 min and stored at −80 °C until experiment. Cells were lysed in lysis buffer [50 mM tris-HCl, 300 mM NaCl, 1 mM EDTA, 1 mM NP-40, 10% glycerol, 1 mM phenylmethylsulfonyl fluoride, and 1× protease inhibitor cocktail (Pierce)] by bead beating with zirconia/silica beads (Biospec) with six cycles of alternating 1-min beating and 2-min pause on ice, using a BioSpec Minibeadbeater, followed by sonication at 50% amplitude for 10 min (30 s on/30 s off cycles) using the EpiShear Multi-Sample Sonicator (Active Motif). Following 15 min of max speed centrifugation, total protein was diluted to a concentration of 1 mg/ml in lysis buffer, denatured with 1× Bolt LDS Sample Buffer and Sample Reducing Agent at 65 °C for 10 min, and applied to a Bolt 4 to 12% Bis-Tris Plus Gel (Thermo Fisher Scientific). The transfer process was performed overnight in Towbin buffer [25 mM tris, 192 mM glycine (pH 8.3), and 20% methanol] at 20 V. Primary antibody was added at the final concentration of 1 mg/ml and incubated overnight at 4 °C, with subsequent secondary antibody (0.1 mg/ml) incubation for 1 h. The Odyssey CLx Scanning System (LI-COR Biosciences) was used for membrane scanning.

### RNA interference and lifespan measurement on *Caenorhabditis elegans*

RNAi plates (standard NGM plates with 25 μg/ml carbenicilli and IPTG 1 mM) were freshly made before beginning of experiment. The worms were maintained for two generation before experiment and moved to RNAi plates at L1 or L4 stage. Lifespan scoring was performed every other day. At least 120 worms were included per RNAi treatment. Worm used in this study is N2. siRNA targeting *hda-6* used in this study is F41H10.6 in Vidal library.

### *Caenorhabditis elegans* RNA collection and purification

Approximately 500 N2 worms at day 3 after RNAi treatment were collected for RNA extraction. The worms were left on hda-6 RNAi Fudr plates when they reach L4 stage, on everyday 3XRNAi food was added to prevent worm from starving. The worms were growing at 20 °C for 3 days and harvested, then lysed in in QIAzol buffer (QIAGEN) with 1.0-mm zirconia/silica beads (BioSpec) for 4 cycles. Total RNA was purified with miRNeasy Mini Kit (QIAGEN). Reverse transcription was performed with the High-Capacity cDNA Reverse Transcription Kit (Thermo Fisher Scientific) using 1 μg of purified RNA.

### *Caenorhabditis elegans* heat treatment

N2 worms were grown on NGM plates with HB109 E. coli containing the empty vector pL4440 (control) and *hda-6* RNAi construct at 20 °C. For each replicate, 32 L4 worms were transferred to fresh plates; when they reached day 1 of adulthood, these worms were incubated in 37 °C. Live worms were counted after 12 h.

### *Caenorhabditis elegans* UV treatment

N2 worms were grown on NGM plates with HB109 E. coli containing the empty vector pL4440 (control) and *hda-6* RNAi construct at 20 °C. Fifty L4 worms were transferred to fresh plates; when they reached day 1 of adulthood, these worms were exposed to 0.08 J/m^2^ UV. Immediately after exposure, worms were transferred to fresh plates and continued to be grown at 20 °C. Live worms were counted and transferred to fresh plates every 2–3 days for the duration of the experiment.

### *Drosophila melanogaster* strains

Flies were reared on standard food at temperature described in the experiment for UAS-Gal4 strains. The following strains were used in this study: tub-Gal4/Tm3, tub-Gal4, tub-Gal80ts/CyO, and HDAC6-RNAi (BL-31053 and BL-34072). All these fly lines were obtained from the Bloomington Drosophila Stock Center.

### *Drosophila melanogaster* adult survival assay

Drosophila embryos and larvae were kept in 18 °C to avoid developmental lethality. Once adult flies emergence, adult male flies were transferred to 25 °C to deactivate Gal80ts, which allows the expression of UAS-*HDAC6* RNAi hairpin to silence the endogenous HDAC6 expression. Adult male flies were then maintained in vials at 25 °C in groups of 10 per vial and passed to a new vial every 3 days. A total of 300 flies were analyzed with 100 flies for each genotype group.

### *Drosophila melanogaster* climbing assay

Climbing male flies was monitored by analyzing their performance to climb 6 cm within 14 s. A successful attempt was scored as 1, and failure to reach the top as 0. Each fly was assessed five times to calculate the average climbing score. 40 flies per age/genotype group were analyzed.

### Chromatin immunoprecipitation and ChIP-seq library preparation

Yeast culture of OD between 0.6 and 0.8 were harvested and cross linked with 37% formaldehyde to final concentration of 1% and incubate at room temperature for 10 min. Cross-linking was stopped by adding 2.5 M glycine to final concentration of 125 mM and incubated at room temperature for 5 min. Cell pellets were resuspend in RIPA buffer (150 mM sodium chloride, 1.0% Triton X-100, 0.5% sodium deoxycholate, 0.1% SDS, 50 mM Tris, pH 8.0), added cold zirconia/silica beads (0.5 mm, BioSpec) and lysed for six rounds. Lysed sample were sonicated for 20 min with Bioruptor, then centrifugated to collect supernatant. Chromatin immunoprecipitation (ChIP) assay was performed using True MicroChip Kit (Diagenode) using designated antibodies listed in Table S3, ChIP DNA are then purified with QIAquick PCR Purification Kit (QIAGEN).

Purified DNA was treated with NEBNext End Repair Module, followed by Agencourt AMPure XP beads (Beckman Coulter) cleanup, and dA (deoxyadenosine) tailing with Klenow fragment (New England BioLabs). After purification, samples were ligated with Illumina TruSeq multiplexing primers using the Quick Ligation Kit (New England BioLabs) and amplified with the KAPA HiFi Ready Mix (Thermo Fisher Scientific). Product was quantified using the Qubit dsDNA HS Assay Kit (Thermo Fisher Scientific). Sequencing was performed on the Illumina HiSeq 2500 or NovaSeq 6000 platform using three biological replicates.

### Yeast strains and media

All strains used in this study excepted for Yeast Knockout collection are listed in Table S4. The yeast knockout collection (mating type alpha) were obtained from Dharmacon (YSC1054). Standard YPD medium (1% yeast extract, 2% peptone, and 2% glucose, with 1.5% agar included for solid medium) was used for all yeast experiments unless otherwise noted.

### Statistics and data processing

#### Statistics

Yeast RLS *p*-values were determined using the Wilcoxon rank-sum test. Statistical significance of all other data not specified above was calculated using an unpaired equal variance two-tailed Student’s *t* test.

#### Barcode sequencing data analysis

Barcode sequencing data were analyzed by a blast-based decode method using a home-made python script. Reads were first trimmed by cutting the 3′ and 5′ adapters. Then trimmed barcodes were aligned using blastn with the following parameters: -task megablast -word_size 7 -reward 2 -penalty -3. Custom blastn database was created by makeblastdb using pre-defined barcode-mutant annotation file. Barcodes that represent specific mutants were counted from the best blastn matches with up to three differences. Uptag and Dntag were counted separately. The count data was then used for the following yeast mutant lifespan estimation.

#### Statistical Model of SEBYL method

We developed a method to estimate yeast mutant lifespan from barcode sequencing counts, with the idea that mutants depleted from the old cell fraction were predicted to have shorter lifespan and those enriched in the old cell fraction were predicted to have a longer lifespan. The barcode counts were generated from nine samples/libraries: the unsorted (USC), young and old cell fraction from the first (S1Y, S1O), second (S2Y, S2O), third (S3Y, S3O), and fourth (S4Y, S4O) of cell sorting.

survival curve of each mutant strain was built by using a modified Gompertz nonlinear regression model (function 1, Fig. 1A).

1 $$S = ae^{ - bc^G}$$

Where *S* is the survival rate estimated from the old cell counts of the mutant strain; *a* is the upper limit of the survival rate, which is 100% here; *e* is Euler’s number; *b* and *c* are two parameters need to be estimated to fit the curve; *G* is the number of generations estimated from the young cell counts of the mutant strain.

Mother cell survival rate (*S*) was calculated using function (2)

2 $${{S}}_{{n}} = {{S}}_{{{n}} - 1} \cdot \frac{{{\mathrm{Fo}}_{{n}} \cdot {\mathrm{To}}_{{n}}}}{{{\mathrm{Fo}}_{{{n}} - 1} \cdot {\mathrm{To}}_{{{n}} - 1} \cdot {{r}}}}$$

Where *n* is the *n*th time of cell sorting (*n* ≤ 3); Fo is the fraction of the mutant in the mother cell sample pool, which is calculated from barcode count data; To is the total cell number of the sample pool, which is manually counted under microscope; *r* is the estimated recovery rate of magnetic beads.

Number of generations (*G*) was calculated by comparing the number of mother cell and daughter cell using function (3)

3 $${{G}}_{{n}} = \log _2\left( {\frac{{{\mathrm{Fy}}_{{n}}}}{{{\mathrm{Fy}}_{{{n}} - 1}}}} \right) + \log _2\left( {\frac{{{\mathrm{Ty}}_{{n}}}}{{{\mathrm{To}}_{{{n}} - 1}}}} \right)$$

Where *n* is the *n*th time of cell sorting (*n* ≤ 3); Fy is the faction of the mutant in the daughter cell sample pool; Ty is the total cell number of the daughter sample pool; To is the total cell number of the mother sample pool;

With the input data of mother cell survival rate and number of growth generations at each time point, parameters *b* and *c* of the model (a) were estimated in R using a nonlinear least squares approach (nls function). After the Gompertz model was fitted, median lifespan was calculated as the number of generations when 50% of the mother cell stop division. P-value for hypothesis of equal trends was calculated by comparing the survival curves between the mutant strain and the wild-type strain using F-test.

#### Identification of traits associated with aging

All morphology data was obtained from yeast morphology database (yeast.gi.k.u-tokyo.ac.jp). Average morphology traits scores of top 10% and bottom 10% long-lived candidates from SEBYL were calculated, Mann–Whitney test was used to determine whether the long-lived and short-lived population have significantly different trait scores, multiple comparison correction was performed with Benjamini–Hochberg procedure.

#### Gene ontology and network analysis

GO analysis of biological process enrichment was performed with the DAVID Functional Annotation Tool (https://david.ncifcrf.gov/), using the GOTERM_BP_DIRECT option. For all GO analyses, default settings were used, and categories with a Benjamini–Hochberg *q* < 0.05 were considered as significantly enriched. Network analysis was performed by STRING database (string-db.org) with all default setting but without text-mining, the results were then exported to cytoscape software (version 3.7.2) for figure generation.

#### RNA-seq data processing

Read alignment was performed using TopHat2 package with the default settings. Read counting and gene expression analysis were performed with htseq-count and edgeR. Transcriptome analysis was based on log2 fold change calculated by edgeR. Significantly up- or down-regulated genes with false discovery rate <0.01 were selected for downstream analysis.

#### ChIP-seq data processing

Read alignment was performed using TopHat2 with the default settings. Subsequent processing was performed using an in-house script where signal extraction scaling^69^ were first applied to normalize each sample for signal scale, samples were then normalized again with trimmed mean of M-values (TMM) method. Metagene plot and heatmap of histone acetylation markers were generated by deeptools package.

### Reporting summary

Further information on research design is available in the Nature Research Reporting Summary linked to this article.

## Supplementary information

Supplementary Information

Supplementary Data 1

Supplementary Data 2

Supplementary Data 3

Supplementary Data 4

Description of Additional Supplementary Files

Reporting Summary

## Data Availability

All high-throughput sequencing data including all RNA-seq and ChIP-seq in this study have been deposited in NCBI BioProject accession PRJNA601478. Additionally, ChIP-seq tracks are also deposited on UCSC genome browser at [http://genome.ucsc.edu/s/chalie102/HDA_ChIP_H3K18ac]. Online database used in this study are: Yeast morphology database [http://scmd.gi.k.u-tokyo.ac.jp/], STRING [https://string-db.org/], Saccharomyces Cerevisiae Database [https://www.yeastgenome.org/]. Source data are provided with this paper.

## Source data

Source data

## Code availability

All codes used in this study are deposited on [https://github.com/Chalietia/].
