# Supplementary material for: Inactivating histone deacetylase HDA promotes longevity by mobilizing trehalose metabolism
Source: Nat Commun. 2021 Mar 31;12:1981. doi: 10.1038/s41467-021-22257-2 (PMC8012573; doi:10.1038/s41467-021-22257-2)
Supplement: Supplementary file 1 — Supplementary Information [file 41467_2021_22257_MOESM1_ESM.docx]

**Inactivating histone deacetylase HDA promotes longevity by mobilizing trehalose metabolism**

**SUPPLEMENTARY INFORMATION**

**Supplementary Figures**

**
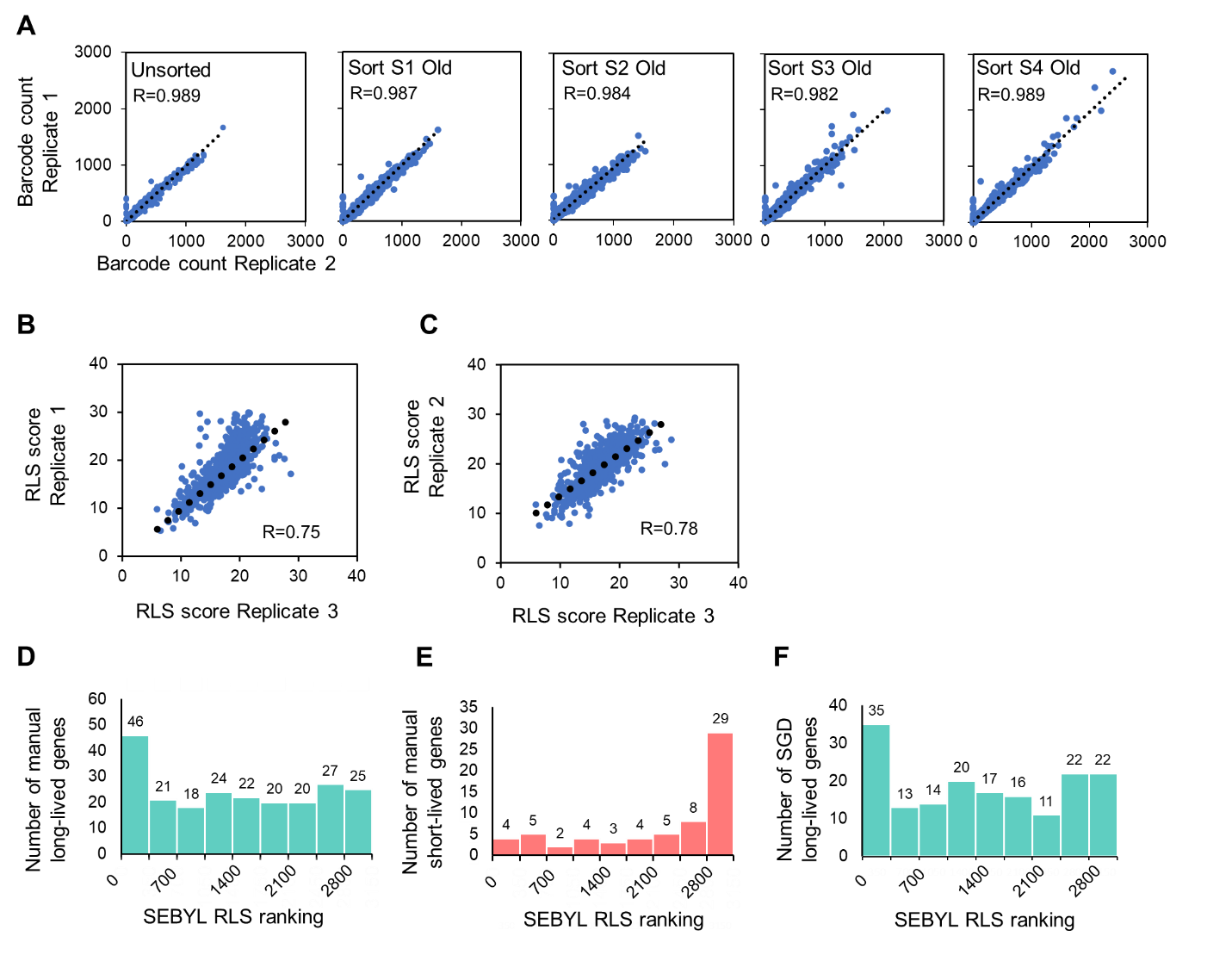
**

**Figure S1. Additional QC and validation on SEBYL screen**

(A) Correlation analysis of barcode counts from screening replicate 1 and replicate 2.

(B) Correlation analysis of final RLS score from screening replicate 1 and replicate 3.

(C) Correlation analysis of final RLS score from screening replicate 2 and replicate 3.

(D) RLS ranking of long-lived deletion strain identified by manual screens (McCormick et al., 2015) in SEBYL results.

(E) RLS ranking of short-lived deletion strain identified by manual screens (McCormick et al., 2015) in SEBYL results.

(F) RLS ranking of long-lived deletion strain listed in SGD database in SEBYL results.


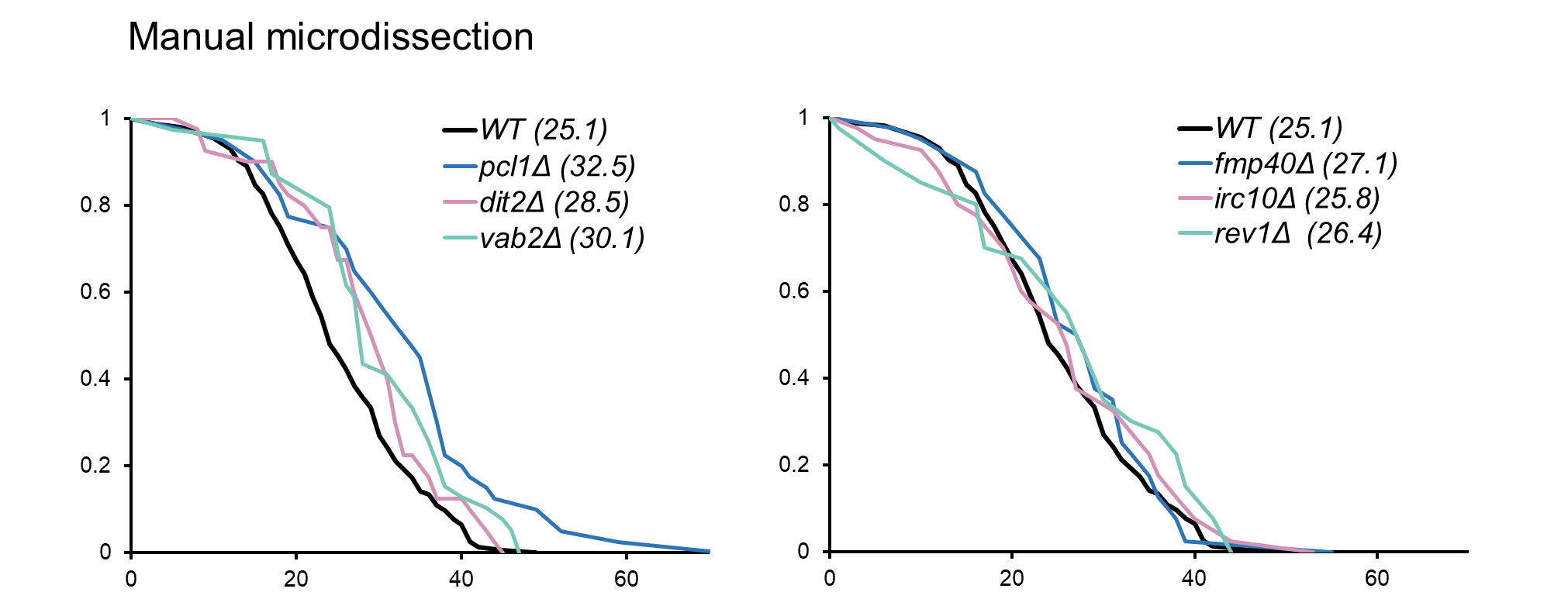


**Figure S2. HDA ablation leads to increase of trehalose metabolism.**

Manual microdissection validation of 6 out of 15 long-lived candidates identified by SEBYL.


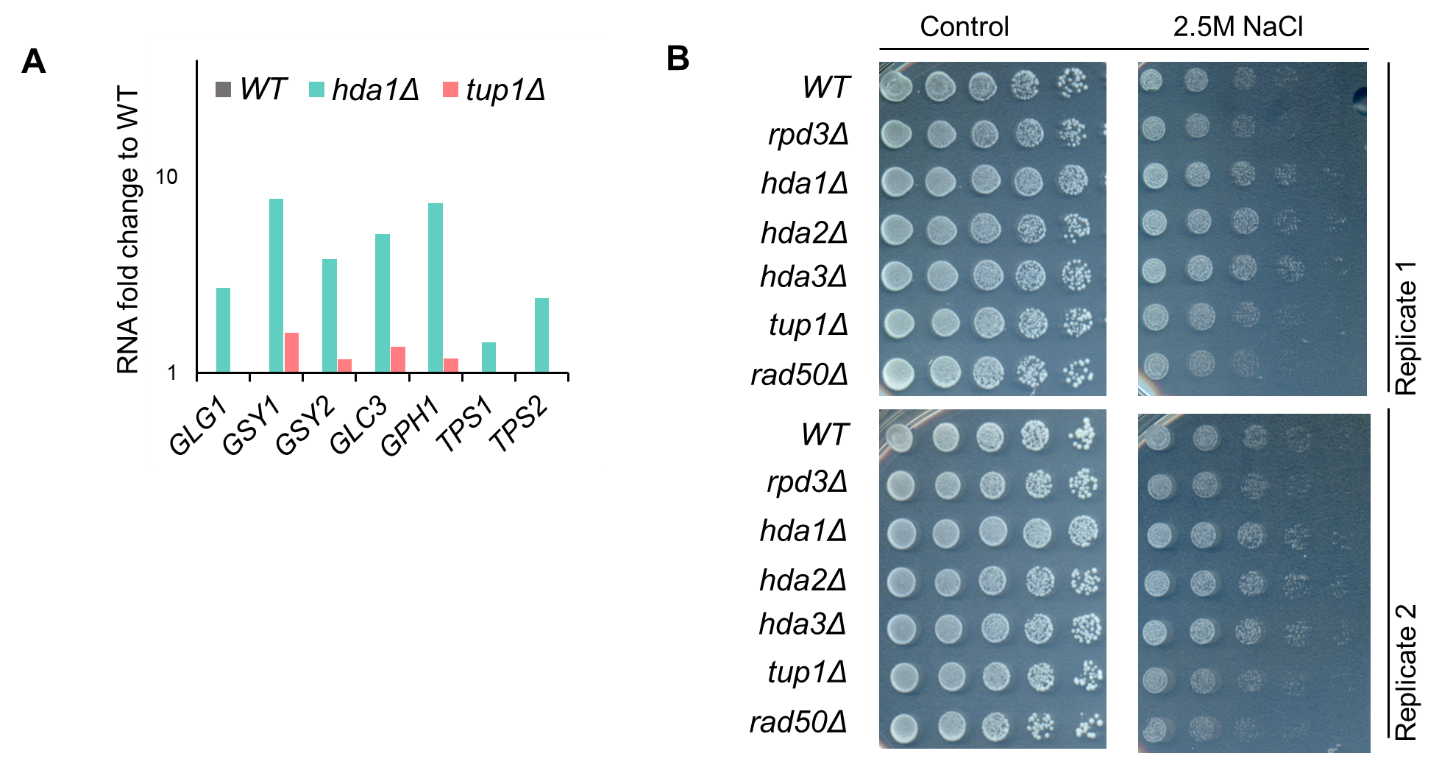


**Figure S3. HDA ablation leads to increase of trehalose metabolism.**

(A) RNA level of designated glycogen and trehalose metabolism genes under MMS treatment. Data from (Gasch et al., 2001).

(B) Growth assay on 2.5M sodium chloride. Control plate used here is synthetic complete (SC) agar plate.


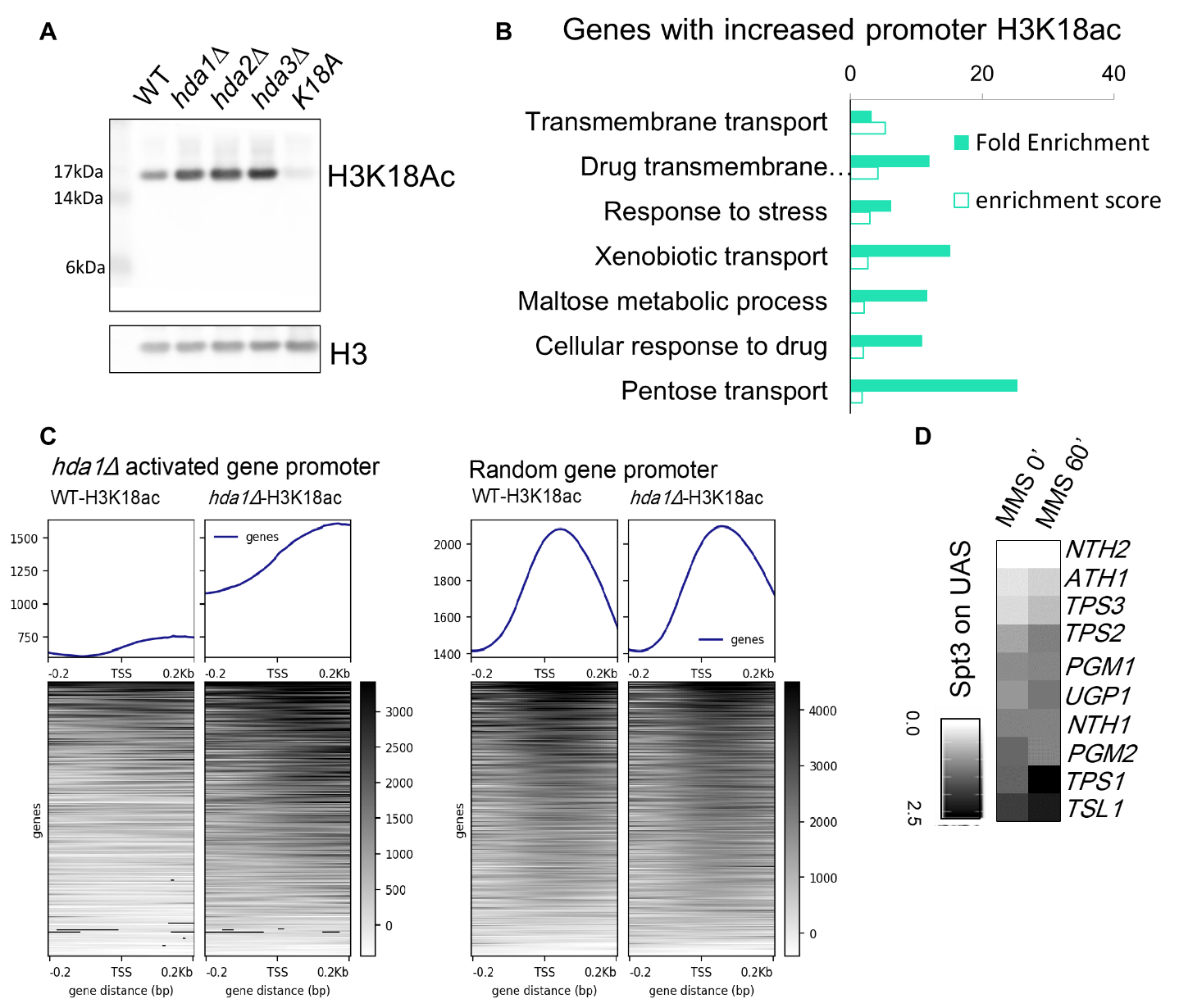


**Figure S4. *HDA* mutation leads to increased H3K18ac level**

(A) Western blot analysis of H3K18 acetylation level in WT, *hda1Δ, hda2Δ, hda3Δ* and H3K18A*.* Experiment was repeated 2 times with similar results.

(B) Gene ontology biology process analysis on genes with increased promoter H3K18 acetylation level. Pathways with Benjamini p value >0.05 were selected.

(C) Metagene plot and heatmap of H3K18 acetylation on genes activated in *hda1Δ* (left) and random genes (right) as detected by ChIP- Seq.

(D) ChIP- chip of Spt3-myc binding on trehalose gene upstream activating sequence (UAS), under 0 or 60 minutes after MMS treatment. Data from (Ghosh, 2011).

**Supplementary Data**

**Supplementary Data1. Results of RLS correlated morphologic trait analysis.**

Supplementary Data1.xlsx

**Supplementary Data2. RLS results from YKO SEBYL screen.**

Supplementary Data2.xlsx

**Supplementary Data3. Statistics of all RLS experiments included in this study.**

Supplementary Data3.xlsx

**Supplementary Data4. List of genes upregulated in *hda1Δ* strain identified by RNA-seq.**

Supplementary Data4.xlsx

**Supplementary Tables**

**Table S1. Developmental lethality of HDAC6-RNAi flies**

|  | **Cross with Tub-Gal4/Tm3,Sb （25°C)** | | |
| --- | --- | --- | --- |
|  | **Short hair**  **(With Tm3)** | **Long hair**  **(With Tub-Gal4)** | **Mortality** |
| W1118 | 246 | 245 | 0.4% |
| HDAC6-RNAi BL-31053 | 247 | 0 | 100.0% |
| HDAC6-RNAi BL-34072 | 252 | 0 | 100.0% |

**Table S2: Oligos used in this study**

| Oligo Name | Oligo Sequence | Reference |
| --- | --- | --- |
| TPS1QF | GTACCCATCTTCCTGAGCGA | This study |
| TPS1QR | GTATGCCAACCACGCATTCT | This study |
| TPS2QF | TGTCACTGTGGGATCTGCAT | This study |
| TPS2QR | CCGTACCAGCACTTTGGAAG | This study |
| GLG1QF | CGATGCAAGAGATGCTACCG | This study |
| GLG1QR | GCATTTCTTGTTGCGGTTGG | This study |
| GSY1QF | GAACGAATGGAAGGCTGACC | This study |
| GSY1QR | TACACAATGGCAGGGCTACA | This study |
| GSY2QF | GATGCTTTAGCAGGCGGAAA | This study |
| GSY2QR | CCTCCTGCAGAGTACCCAAA | This study |
| GLC3QF | TTGAAACCGTTTGCCGATGT | This study |
| GLC3QR | CTTTGGTTTCCGGATTGGCA | This study |
| TPS1-promoterQF | TTGAACAAGCACGCAGCTAA | This study |
| TPS1-promoterQR | CCCTGAAGACGAGGTCAGTT | This study |
| NTH1-promoter-f | ACTGATTTCACAACCAACTGC | This study |
| NTH1-promoter-R | CTACCGGTCCTTGGCTTGTA | This study |
| iYHL004W F | GCCAACCCGAAGACGCTAAA | Park et. al. |
| iYHL004W R | AGAAGCATAAACCCAAGTACGAA | Park et. al. |
| ACT1 Forward | TCGTTCCAATTTACGCTGGTT | This study |
| ACT1 Reverse | CGGCCAAATCGATTCTCAA | This study |
| tps1-wormF | GCCAGCACTTCACAATTTGC | This study |
| tps1-wormR | ACACGTACATAGGCACACCA | This study |
| tps2-wormF | CGCGTACACACGAATCTCTC | This study |
| tps2-wormR | CCCATGTGAATGGACGGAAC | This study |
| tre1-wormF | GCAGAGCATCTGCAAACTGA | This study |
| tre1-wormR | GCGTTTAAATCGGCTGGGAT | This study |
| tre2-wormF | ATCATGCCACAAGCTTCCAC | This study |
| tre2-wormR | GCTGATGGCTCCAGATGTTG | This study |
| tre3-wormF | TATGGGCAACTTCCGAGTGT | This study |
| tre3-wormR | AGATCCTGGTTGTGGGTAGC | This study |
| tre4-wormF | GCGCTTGGTTTGACTACGAT | This study |
| tre4-wormR | GAATACCGCCCACAAACTCC | This study |
| tre5-wormF | TTCTACGTACCGGCTCGAAA | This study |
| tre5-wormR | GAATTCCGCCGGGTATTGAG | This study |

**Table S3: Antibodies used in this study**

| Antibody | Source | Identifier | Lot | Dilution |
| --- | --- | --- | --- | --- |
| Mouse anti histone H3 | Active Motif | 61475 | 17316008 | 1:1000 |
| Rabbit anti H3k18ac | Active Motif | 39587 | 07909001 | 1:1000 |
| Mouse anti c-Myc | Santa Cruz | sc-40 | L1510-9E10 | 1:500 |
| Goat anti-Mouse IgG Secondary Antibody, DyLight 800 | Thermofisher | 35521 | OC183251 | 1:5000 |
| Goat anti-Rabbit IgG Secondary Antibody, DyLight 680 | Thermofisher | 35568 | NL181279 | 1:5000 |
| Mouse anti GAPDH | Thermofisher | MA515738 | OG187837 | 1:5000 |

**Table S4: All yeast strains used in this study**

| Strain Name | Genotype | Source |
| --- | --- | --- |
| BY4741 | *MATa his3∆1 leu2∆0 met15∆0 ura3∆0* | Thermo Fisher |
| BY4742 | *MATα his3Δ1 leu2Δ0 lys2Δ0 ura3Δ0* | Thermo Fisher |
| YKO1706 | *BY4741 irc10∆::KANMX4* | Thermo Fisher |
| YKO1643 | *BY4741 rev1Δ::KANMX4* | Thermo Fisher |
| YKO1740 | *BY4741 gsh2Δ::KANMX4* | Thermo Fisher |
| YKO1070 | *BY4741 fmp40Δ::KANMX4* | Thermo Fisher |
| YKO7260 | *BY4741 mfa1Δ::KANMX4* | Thermo Fisher |
| YKO1155 | *BY4741 pcl1Δ::KANMX4* | Thermo Fisher |
| YKO1693 | *BY4741 izh2Δ::KANMX4* | Thermo Fisher |
| YKO4238 | *BY4741 dit2Δ::KANMX4* | Thermo Fisher |
| YKO245 | *BY4741 vab2Δ::KANMX4* | Thermo Fisher |
| YKO5913 | *BY4741 apl6Δ::KANMX4* | Thermo Fisher |
| YKO6819 | *BY4741 rfc107Δ::KANMX4* | Thermo Fisher |
| YKO3855 | *BY4741 ydl157cΔ::KANMX4* | Thermo Fisher |
| YKO5347 | *BY4741 hda1Δ::KANMX4* | Thermo Fisher |
| YKO3201 | *BY4741 ybr064wΔ::KANMX4* | Thermo Fisher |
| YKO2695 | *BY4741 pax2Δ::KANMX4* | Thermo Fisher |
| YKO11706 | *BY4742 irc10∆::KANMX4* | Thermo Fisher |
| YKO11643 | *BY4742 rev1Δ::KANMX4* | Thermo Fisher |
| YKO11740 | *BY4742 gsh2Δ::KANMX4* | Thermo Fisher |
| YKO11070 | *BY4742 fmp40Δ::KANMX4* | Thermo Fisher |
| YKO17260 | *BY4742 mfa1Δ::KANMX4* | Thermo Fisher |
| YKO11155 | *BY4742 pcl1Δ::KANMX4* | Thermo Fisher |
| YKO11693 | *BY4742 izh2Δ::KANMX4* | Thermo Fisher |
| YKO14238 | *BY4742 dit2Δ::KANMX4* | Thermo Fisher |
| YKO10245 | *BY4742 vab2Δ::KANMX4* | Thermo Fisher |
| YKO15913 | *BY4742 apl6Δ::KANMX4* | Thermo Fisher |
| YKO16819 | *BY4742 rfc107Δ::KANMX4* | Thermo Fisher |
| YKO13855 | *BY4742 ydl157cΔ::KANMX4* | Thermo Fisher |
| YKO15347 | *BY4742 hda1Δ::KANMX4* | Thermo Fisher |
| YKO13201 | *BY4742 ybr064wΔ::KANMX4* | Thermo Fisher |
| YKO12695 | *BY4742 pax2Δ::KANMX4* | Thermo Fisher |
| YKO5594 | *BY4741 hda3Δ::KANMX4* | Thermo Fisher |
| YKO7198 | *BY4741 tup1Δ::KANMX4* | Thermo Fisher |
| RYY200 | *BY4741 hda1Δ::HYGMX4 tup1Δ::KANMX4* | This study |
| YKO6464 | *BY4741 rad50Δ::KANMX4* | Thermo Fisher |
| YKO5167 | *BY4741 gsy2Δ::KANMX4* | Thermo Fisher |
| YKO5575 | *BY4741 gph1Δ::KANMX4* | Thermo Fisher |
| YKO3265 | *BY4741 tps3Δ::KANMX4* | Thermo Fisher |
| YKO3941 | *BY4741 nth1Δ::KANMX4* | Thermo Fisher |
| RYY201 | *BY4741 hda1Δ::HYGMX4 gsy2Δ::KANMX4* | This study |
| RYY202 | *BY4741 hda1Δ::HYGMX4 gph1Δ::KANMX4* | This study |
| RYY203 | *BY4741 hda1Δ::HYGMX4 tps3Δ::KANMX4* | This study |
| RYY204 | *BY4741 hda1Δ::HYGMX4 nth1Δ::KANMX4* | This study |
| Msy196_H3K9Q | *MATalpha his3∆ leu2∆ ura3∆ hht1(K9Q)-hhf1::HHTS-HHFS-HygMX4 hht2(K9Q)-hhf2::HHTS-HHFS-URS3 can1::STE2pr-spHIS5 lyp1::STE3pr-LEU2* | Dai et. Al. |
| RYY205 | *MATalpha his3∆ leu2∆ ura3∆ hht1(K9Q)-hhf1::HHTS-HHFS-HygMX4 hht2(K9Q)-hhf2::HHTS-HHFS-URS3 can1::STE2pr-spHIS5 lyp1::STE3pr-LEU2 hda1∆::KANMX4* | This study |
| Msy196_H3K14Q | *MATalpha his3∆ leu2∆ ura3∆ hht1(K14Q)-hhf1::HHTS-HHFS-HygMX4 hht2(K14Q)-hhf2::HHTS-HHFS-URS3 can1::STE2pr-spHIS5 lyp1::STE3pr-LEU2* | Dai et. Al. |
| RYY206 | *MATalpha his3∆ leu2∆ ura3∆ hht1(K14Q)-hhf1::HHTS-HHFS-HygMX4 hht2(K14Q)-hhf2::HHTS-HHFS-URS3 can1::STE2pr-spHIS5 lyp1::STE3pr-LEU2 hda1∆::KANMX4* | This study |
| Msy196_H3K18Q | *MATalpha his3∆ leu2∆ ura3∆ hht1(K18Q)-hhf1::HHTS-HHFS-HygMX4 hht2(K18Q)-hhf2::HHTS-HHFS-URS3 can1::STE2pr-spHIS5 lyp1::STE3pr-LEU2* | Dai et. Al. |
| RYY207 | *MATalpha his3∆ leu2∆ ura3∆ hht1(K18Q)-hhf1::HHTS-HHFS-HygMX4 hht2(K18Q)-hhf2::HHTS-HHFS-URS3 can1::STE2pr-spHIS5 lyp1::STE3pr-LEU2 hda1∆::KANMX4* | This study |
| Msy196_H3K23Q | *MATalpha his3∆ leu2∆ ura3∆ hht1(K23Q)-hhf1::HHTS-HHFS-HygMX4 hht2(K23Q)-hhf2::HHTS-HHFS-URS3 can1::STE2pr-spHIS5 lyp1::STE3pr-LEU2* | Dai et. Al. |
| RYY208 | *MATalpha his3∆ leu2∆ ura3∆ hht1(K23Q)-hhf1::HHTS-HHFS-HygMX4 hht2(K23Q)-hhf2::HHTS-HHFS-URS3 can1::STE2pr-spHIS5 lyp1::STE3pr-LEU2 hda1∆::KANMX4* | This study |
| Msy196_H3K27Q | *MATalpha his3∆ leu2∆ ura3∆ hht1(K27Q)-hhf1::HHTS-HHFS-HygMX4 hht2(K27Q)-hhf2::HHTS-HHFS-URS3 can1::STE2pr-spHIS5 lyp1::STE3pr-LEU2* | Dai et. Al. |
| RYY209 | *MATalpha his3∆ leu2∆ ura3∆ hht1(K27Q)-hhf1::HHTS-HHFS-HygMX4 hht2(K27Q)-hhf2::HHTS-HHFS-URS3 can1::STE2pr-spHIS5 lyp1::STE3pr-LEU2 hda1∆::KANMX4* | This study |
| Msy196_H3K18R | *MATalpha his3∆ leu2∆ ura3∆ hht1(K18R)-hhf1::HHTS-HHFS-HygMX4 hht2(K18R)-hhf2::HHTS-HHFS-URS3 can1::STE2pr-spHIS5 lyp1::STE3pr-LEU2* | Dai et. Al. |
| RYY210 | MATalpha his3∆ leu2∆ ura3∆ hht1(K18R)-hhf1::HHTS-HHFS-HygMX4 hht2(K18R)-hhf2::HHTS-HHFS-URS3 can1::STE2pr-spHIS5 lyp1::STE3pr-LEU2 hda1∆::KANMX4 | This study |
| RYY211 | BY4741 SPT3-13myc::HIS3 | This study |
| RYY212 | BY4741 *tps2Δ::KANMX4* | This study |
| RYY213 | BY4741 *tps2Δ::KANMX4 hda1Δ::URA3* | This study |
