# Supplementary material for: Inactivating histone deacetylase HDA promotes longevity by mobilizing trehalose metabolism
Source: Nat Commun. 2021 Mar 31;12:1981. doi: 10.1038/s41467-021-22257-2 (PMC8012573; doi:10.1038/s41467-021-22257-2)
Supplement: Supplementary file 6 — Description of Additional Supplementary Files [file 41467_2021_22257_MOESM6_ESM.docx]

Description of Additional Supplementary Information

Title: Supplementary Data 1.

Description: Results of RLS correlated morphologic trait analysis.

Title: Supplementary Data 2.

Description: RLS results from YKO SEBYL screen.

Title: Supplementary Data 3.

Description: Statistics of all RLS experiments included in this study.

Title: Supplementary Data 4.

Description: List of genes upregulated in hda1Δ strain identified by RNA-seq.
