# Supplementary material for: Inactivating histone deacetylase HDA promotes longevity by mobilizing trehalose metabolism
Source: Nat Commun. 2021 Mar 31;12:1981. doi: 10.1038/s41467-021-22257-2 (PMC8012573; doi:10.1038/s41467-021-22257-2)
Supplement: Supplementary file 7 — Reporting Summary [file 41467_2021_22257_MOESM7_ESM.pdf]

nature research

Corresponding author(s): Weiwei Dang

Last updated by author(s): 02/14/2021

Reporting Summary

Nature Research wishes to improve the reproducibility of the work that we publish. This form provides structure for consistency and transparency in reporting. For further information on Nature Research policies, see our Editorial Policies and the Editorial Policy Checklist.

Statistics

For all statistical analyses, confirm that the following items are present in the figure legend, table legend, main text, or Methods section.

n/a	Confirmed	
☐	☒	The exact sample size (*n*) for each experimental group/condition, given as a discrete number and unit of measurement
☐	☒	A statement on whether measurements were taken from distinct samples or whether the same sample was measured repeatedly
☐	☒	The statistical test(s) used AND whether they are one- or two-sided<br>*Only common tests should be described solely by name; describe more complex techniques in the Methods section.*
☐	☒	A description of all covariates tested
☐	☒	A description of any assumptions or corrections, such as tests of normality and adjustment for multiple comparisons
☐	☒	A full description of the statistical parameters including central tendency (e.g. means) or other basic estimates (e.g. regression coefficient) AND variation (e.g. standard deviation) or associated estimates of uncertainty (e.g. confidence intervals)
☐	☒	For null hypothesis testing, the test statistic (e.g. F, t, r) with confidence intervals, effect sizes, degrees of freedom and P value noted<br>*Give P values as exact values whenever suitable.*
☒	☐	For Bayesian analysis, information on the choice of priors and Markov chain Monte Carlo settings
☒	☐	For hierarchical and complex designs, identification of the appropriate level for tests and full reporting of outcomes
☐	☒	Estimates of effect sizes (e.g. Cohen's *d*, Pearson's *r*), indicating how they were calculated

Our web collection on statistics for biologists contains articles on many of the points above.

Software and code

Policy information about availability of computer code

Data collection	Trehalose and Glycogen measurement: Data was collected with Gen5 software version 3.0 ; All microfluid experiments image were recorded with EVOS FL Auto Cell Imaging System Software version 2.0.2094.0.
Data analysis	qPCR data analysis was performed by QuantStudio 7 Real-Time PCR software (version 1.3);trehalose and Glycogen measurement: OD data was processed with Gen5 software (Version 3.0); GO analysis as performed with DAVID Functional Annotation Tool (https://david.ncifcrf.gov/); Read alignment of RNA-seq and ChIP-seq was performed with TopHat2 (Version 2.1.1); Transcriptome analysis was performed with htseq (0.11.1) and edgeR (Version 3.32.1); Metagene plot and heatmap of histone acetylation markers were generated by deeptools package (Version 3.5.0); Gene network figure was generated by Cytoscape (3.4.0); ChIP-seq and barcode sequencing data were analyzed with in-house scripts that has been deposited at https://github.com/Chalietia/ .

For manuscripts utilizing custom algorithms or software that are central to the research but not yet described in published literature, software must be made available to editors and reviewers. We strongly encourage code deposition in a community repository (e.g. GitHub). See the Nature Research guidelines for submitting code & software for further information.

Data

Policy information about availability of data

All manuscripts must include a data availability statement. This statement should provide the following information, where applicable:

- Accession codes, unique identifiers, or web links for publicly available datasets
- A list of figures that have associated raw data
- A description of any restrictions on data availability

All high-throughput sequencing data in this study have been deposited in NCBI BioProject accession PRJNA601478 and http://genome.ucsc.edu/s/chalie102/HDA_ChIP_H3K18ac. Other resources are available upon request.

Field-specific reporting

Please select the one below that is the best fit for your research. If you are not sure, read the appropriate sections before making your selection.

☒ Life sciences ☐ Behavioural & social sciences ☐ Ecological, evolutionary & environmental sciences

For a reference copy of the document with all sections, see nature.com/documents/nr-reporting-summary-flat.pdf

Life sciences study design

All studies must disclose on these points even when the disclosure is negative.

Sample size	No sample size calculation was performed. Sample size was determined according to previous literature.
Data exclusions	In figure 4J, 3 data points for 4,6,8 hrs were removed due to aberrantly high OD value. Since data from 10-34 hours were in a consistent range, these afore-mentioned three data points were removed from both experiments.
Replication	Replicate numbers for individual experiments were listed in figure legends or supplementary. Briefly, all NGS experiments have at least three biological replicates, all qPCR experiments have at least three technical replicates, growth curve OD measurement have four technical replicates for each genotype. All attempts on replication were successful.
Randomization	No randomization was performed. All strains used in this study is isogenic and are therefore considered identical, any genetic manipulation and chemical treatment is therefore intrinsically randomized.
Blinding	Not relevant in this study, all experiment outcomes are objective and not likely subjected to bias.

Reporting for specific materials, systems and methods

We require information from authors about some types of materials, experimental systems and methods used in many studies. Here, indicate whether each material, system or method listed is relevant to your study. If you are not sure if a list item applies to your research, read the appropriate section before selecting a response.

Materials & experimental systems

n/a	Involved in the study	
☐	☒	Antibodies
☒	☐	Eukaryotic cell lines
☒	☐	Palaeontology and archaeology
☐	☒	Animals and other organisms
☒	☐	Human research participants
☒	☐	Clinical data
☒	☐	Dual use research of concern

Methods

n/a	Involved in the study	
☐	☒	ChIP-seq
☒	☐	Flow cytometry
☒	☐	MRI-based neuroimaging

Antibodies

Antibodies used	Mouse anti histone H3 Active Motif 61475<br>Rabbit anti H3k18ac Active Motif 39587<br>Mouse anti c-Myc Santa Cruz sc-40<br>Goat anti-Mouse IgG Secondary Antibody, DyLight 800 Thermofisher 35521<br>Goat anti-Rabbit IgG Secondary Antibody, DyLight 680 Thermofisher 35568<br>Mouse anti GAPDH Thermofisher MA515738
Validation	Active Motif 61475: Validated for Western blot and ChIP-Seq by manufacturer<br>Active Motif 39587: Validated for Western blot and ChIP by manufacturer<br>Santa Cruz sc-40: Validated for Western blot and Immunostaining by manufacturer<br>Thermofisher MA515738: Validated for Western blot and Immunostaining by manufacturer

Animals and other organisms

Policy information about studies involving animals; ARRIVE guidelines recommended for reporting animal research

Laboratory animals	C. elegans, lab strain N2L4440, hermaphrodite; D. melanogaster: strain tub-Gal4/Tm3, tub-Gal4, tub-Gal80ts/CyO, male.
Wild animals	NA

Field-collected samples: NA

Ethics oversight: No IRB or IACUC approval is required for our study using laboratory yeast, worms and flies.

Note that full information on the approval of the study protocol must also be provided in the manuscript.

ChIP-seq

Data deposition

☒ Confirm that both raw and final processed data have been deposited in a public database such as GEO.

☒ Confirm that you have deposited or provided access to graph files (e.g. BED files) for the called peaks.

Data access links
May remain private before publication.

https://www.ncbi.nlm.nih.gov/bioproject/PRJNA601478

Files in database submission

[RNA-Seq]WT rep1
[RNA-Seq]WT rep2
[RNA-Seq]WT rep3
[RNA-Seq]hda1 deletion rep1
[RNA-Seq]hda1 deletion rep2
[RNA-Seq]hda1 deletion rep3
[ChIP-Seq against total H3 protein]WT rep1
[ChIP-Seq against total H3 protein]WT rep2
[ChIP-Seq against total H3 protein]WT rep3
[ChIP-Seq against total H3 protein]hda1d rep1
[ChIP-Seq against total H3 protein]hda1d rep2
[ChIP-Seq against total H3 protein]hda1d rep3
[ChIP-Seq against H3K18acetylation]WT rep1
[ChIP-Seq against H3K18acetylation]WT rep2
[ChIP-Seq against H3K18acetylation]WT rep3
[ChIP-Seq against H3K18acetylation]hda1d rep1
[ChIP-Seq against H3K18acetylation]hda1d rep2
[ChIP-Seq against H3K18acetylation]hda1d rep3

Genome browser session
(e.g. UCSC)

http://genome.ucsc.edu/s/chalie102/HDA_ChIP_H3K18ac

Methodology

Replicates: all NGS experiments have three replicates each

Sequencing depth:

wt-H3_1 wt-H3_2 wt-H3_3 hda-H3_1 hda-H3_2 hda-H3_3 WT-K18_1 WT-K18_2 WT-K18_3 hda-K18_1 hda-K18_2 hda-K18_3
Total_reads 28603606 + 0 in total (QC-passed reads + QC-failed reads) 31257192 + 0 in total (QC-passed reads + QC-failed reads) 31681122 + 0 in total (QC-passed reads + QC-failed reads) 24703952 + 0 in total (QC-passed reads + QC-failed reads) 27276666 + 0 in total (QC-passed reads + QC-failed reads) 26536648 + 0 in total (QC-passed reads + QC-failed reads) 34360904 + 0 in total (QC-passed reads + QC-failed reads) 31966472 + 0 in total (QC-passed reads + QC-failed reads) 36049924 + 0 in total (QC-passed reads + QC-failed reads) 26130252 + 0 in total (QC-passed reads + QC-failed reads) 24836488 + 0 in total (QC-passed reads + QC-failed reads) 25456922 + 0 in total (QC-passed reads + QC-failed reads)
UniqueMapped 27533672 + 0 mapped (96.26% : N/A) 30017714 + 0 mapped (96.03% : N/A) 30124036 + 0 mapped (95.09% : N/A) 23060795 + 0 mapped (93.35% : N/A) 25592390 + 0 mapped (93.83% : N/A) 24738643 + 0 mapped (93.22% : N/A) 32434859 + 0 mapped (94.39% : N/A) 30546526 + 0 mapped (95.56% : N/A) 34530337 + 0 mapped (95.78% : N/A) 23585508 + 0 mapped (90.26% : N/A) 22453398 + 0 mapped (90.40% : N/A) 23059026 + 0 mapped (90.58% : N/A)
Read_Length ~100bp ~100bp ~100bp ~100bp ~100bp ~100bp ~100bp ~100bp ~100bp ~100bp ~100bp ~100bp
Pair_or_single Paired Paired Paired Paired Paired Paired Paired Paired Paired Paired Paired Paired

Antibodies:
Mouse anti histone H3 Active Motif 61475
Rabbit anti H3k18ac Active Motif 39587

Peak calling parameters: Due to the relatively low enrichment of H3K18ac no peak calling was performed.

Data quality: Due to the relatively low enrichment of H3K18ac no peak calling was performed.

Software: Read alignment was performed using TopHat2 with the default settings. Subsequent processing was performed using an in-house script where signal extraction scaling (Diaz et al., 2012) were first applied to normalize each sample for signal scale, samples were then normalized again with trimmed mean of M-values (TMM) method. Script was deposited on https://github.com/Chalietia/ChIPSES
