# Supplementary material for: Inactivating histone deacetylase HDA promotes longevity by mobilizing trehalose metabolism
Source: Nat Commun. 2021 Mar 31;12:1981. doi: 10.1038/s41467-021-22257-2 (PMC8012573; doi:10.1038/s41467-021-22257-2)
Supplement: Supplementary file 8 — Source data [file 41467_2021_22257_MOESM8_ESM.zip › SupFig4A_original_blot2.docx]

H3

H3K18Ac
